# Recent Advances in Low-Dimensional Metal Oxides via Sol-Gel Method for Gas Detection

**DOI:** 10.3390/nano14040359

**Published:** 2024-02-14

**Authors:** Marwa Ben Arbia, Hicham Helal, Elisabetta Comini

**Affiliations:** Sensor Lab, Department of Information Engineering, University of Brescia, Via Valotti 9, 25133 Brescia, Italy; m.benarbia@studenti.unibs.it (M.B.A.); h.helal@unibs.it (H.H.)

**Keywords:** Sol-Gel, metal oxides, nanostructures, low dimensional, gas sensors

## Abstract

Low-dimensional metal oxides have drawn significant attention across various scientific domains due to their multifaceted applications, particularly in the field of environment monitoring. Their popularity is attributed to a constellation of unique properties, including their high surface area, robust chemical stability, and remarkable electrical conductivity, among others, which allow them to be a good candidate for detecting CO, CO_2_, H_2_, NH_3_, NO_2_, CH_4_, H_2_S, and volatile organic compound gases. In recent years, the Sol-Gel method has emerged as a powerful and versatile technique for the controlled synthesis of low-dimensional metal oxide materials with diverse morphologies tailored for gas sensing applications. This review delves into the manifold facets of the Sol-Gel processing of metal oxides and reports their derived morphologies and remarkable gas-sensing properties. We comprehensively examine the synthesis conditions and critical parameters governing the formation of distinct morphologies, including nanoparticles, nanowires, nanorods, and hierarchical nanostructures. Furthermore, we provide insights into the fundamental principles underpinning the gas-sensing mechanisms of these materials. Notably, we assess the influence of morphology on gas-sensing performance, highlighting the pivotal role it plays in achieving exceptional sensitivity, selectivity, and response kinetics. Additionally, we highlight the impact of doping and composite formation on improving the sensitivity of pure metal oxides and reducing their operation temperature. A discussion of recent advances and emerging trends in the field is also presented, shedding light on the potential of Sol-Gel-derived nanostructures to revolutionize the landscape of gas sensing technologies.

## 1. Introduction

In recent years, detecting hazardous gases has been a major challenge in many applications, primarily focused on ensuring the safety of working and living spaces. Locations like these often contain dangerous gases such as carbon monoxide (CO) and volatile organic compounds (VOCs) such as formaldehyde. The former emanates from inadequate oxygen levels during coal combustion or gases in heating devices. At the same time, the latter is a byproduct of indoor furniture, often constructed with urea, melamine, or phenol to produce thermosetting resins used in coatings, adhesives, and foams. The surveillance of indoor air quality is essential, particularly in cases where the detected gases are not highly dangerous or toxic. Detecting and eliminating these gases can greatly improve the overall air quality. Sensors also have important roles in chemical plants, such as in reactors, pipelines, and furnaces, where it is vital to continuously monitor atmospheric conditions to ensure that the processes remain within specified limits. Furthermore, in such environments, sensors are employed to cleanse exhaust gases of harmful compounds before they are discharged into the environment [1,2].

Sensing devices consist of two primary components: a receptor responsible for converting chemical information into a quantifiable energy form, and a transducer that converts this energy into a signal. When the receptor interacts with the analyte, the sensor is categorized as a ‘chemical sensor’ [3]. If this interaction involves biochemical components, the sensor is commonly referred to a ‘biochemical sensor’ [4,5]. In cases where there is no chemical reaction, and the interaction between the analyte and the receptor results in a change in certain physical properties such as mass, temperature, electrical characteristics, magnetism, or optical attributes, the sensor is labeled as a ‘physical sensor’ [6].

The fundamental operational concept of a gas sensor (as a physical sensor) involves a reversible change in the electrical conductivity resulting from the interaction between the sensor and gaseous substances in the surrounding environment, both before and after the latter are adsorbed. These reactions at the sensing layer’s surface leads to the generation of an electric output signal [7].

At present, a range of gas sensors has been created by researchers using different technologies: catalytic gas sensors, electrochemical gas sensors, thermal conductivity gas sensors, optical gas sensors, acoustic gas sensors, and semiconductor sensors including metal oxide semiconductors (MOXs) [8]. The MOX gas sensor has gained great attention due to its high sensitivity, cost-effective production, direct measurement, and signal characteristics.

For many years, solid-state chemists have conducted extensive research on MOXs [9]. Nevertheless, traditional solid-state synthesis methods involving the reaction of powdered precursors are inadequate for producing nanoscale metal oxides with precisely defined characteristics in terms of shape, size, and composition. In contrast to these high-temperature procedures, soft-chemistry approaches, specifically Sol-Gel techniques, offer distinct advantages. They allow for the creation of metastable materials, ensuring heightened purity and compositional consistency in the resulting products at relatively moderate temperatures using standard laboratory equipment. Additionally, they permit the manipulation of particle morphology during the chemical conversion of the molecular precursor into the ultimate oxide structure [10,11].

**Figure 1:**
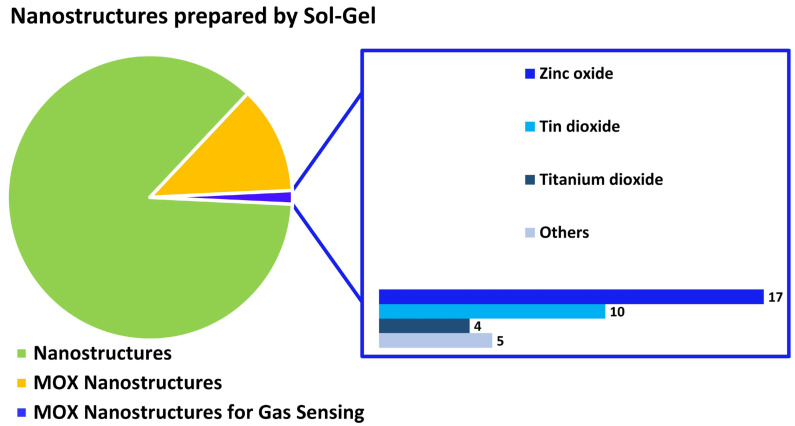
Publications on nanostructures prepared by Sol–Gel within 2018–2023 from Web of Science using the search words ‘Sol-Gel’, ‘Nanostructures’, ‘Metal oxides’, ‘Gas sensing’, 31 January 2024.

Sol-Gel processing can yield a diverse range of low-dimensional structures, such as nanowires, nanoneedles, hexagons, nanorods, nanoribbons, nanobelts, nanoflakes, nanoparticles, nanosheets, nanoplates, and hierarchical structures [12,13]. These capabilities prompt researchers to prepare Sol-Gel-derived MOX with high specific surface areas, leading to enhancing the gas adsorption and surface interactions for improved gas detection. Figure 1 presents a pie chart showing the number of publications on nanostructures obtained via Sol-Gel, with a specific focus on metal oxide semiconductors for sensing applications in the last five years.

This review aims to comprehensively explore recent advancements in the synthesis and application of low-dimensional metal oxides for gas detection, specifically focusing on the Sol-Gel method. It provides an in-depth overview of the latest developments in the field, emphasizing the synthesis of metal oxides through the Sol-Gel approach. The review evaluates the performance and potential applications of these materials in gas detection, with a focus on sensitivity, selectivity, and overall effectiveness. Additionally, it details various methodologies employed in the Sol-Gel process to fabricate low-dimensional metal oxides, highlighting key parameters influencing their properties. The primary focus of this review is on studies published within the last decade, ensuring a contemporary examination of the field that captures the most recent developments and trends.

The content of this manuscript presents a brief history of the Sol-Gel method and its basic steps and parameters influencing the morphology of the prepared materials. Then, Sol-Gel-derived metal oxides and their morphologies are introduced for possible gas sensing applications. This review also provides an overview of gas sensing mechanisms and properties for both n-type and p-type metal oxides, shedding light on doping and composite formation for optimizing the gas responsiveness of metal oxides.

By presenting a structured review with these objectives, the aim is to offer a clear and valuable resource for researchers and practitioners interested in the Sol-Gel synthesis of low-dimensional metal oxides for gas detection.

## 2. Sol-Gel Processing

### 2.1. Brief History

The pioneering discovery of the Sol-Gel method is credited to Ebelmen, who first reported it in 1846 while exploring the production of silica glass [14]. The 19th century witnessed significant progress in colloidal chemistry, which paved the way for modern Sol-Gel science. In 1861, Michael Faraday described the optical properties of gold and silver nanoparticles, acknowledging the existence of colloidal solutions. Later, in 1871, John Tyndall conducted studies on light scattering by colloidal particles, providing further insights into the behavior of colloids: When a beam of light traverses through a colloid, revealing a distinct bright ‘path’ within the colloid, aligned with the direction of the incident light, the colloid becomes apparent. This phenomenon is known as the Faraday–Tyndall effect which allows colloids to be distinguished from solutions [15].

**Figure 2:**
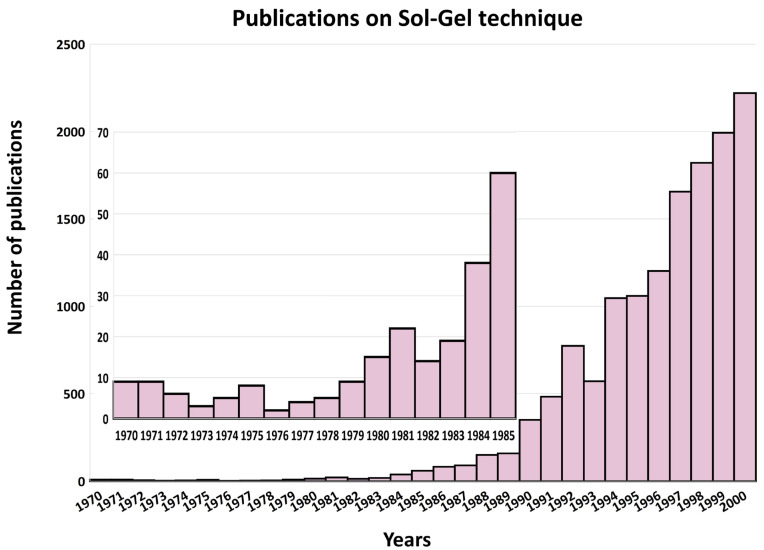
Number of publications on Sol-Gel method for the period of 1970–2000 (inset: zoom on the publication number in the period 1970–1985) from Web of Science using the search word ‘Sol-Gel’, 31 January 2024.

The early 20th century saw the emergence of Sol-Gel science as a distinct field of study. The development of Sol-Gel ceramics gained momentum, with researchers exploring the synthesis of various metal oxide materials with tailored properties. In the 1970s and 1980s, the Sol-Gel method found numerous applications beyond glass and ceramics. It was recognized as an innovative approach for producing homogeneous materials at reduced temperatures, distinct from the traditional melting and sintering methods [16]. Researchers started using the process to create thin films, coatings, and fibers with unique properties. Since the late 20th century and into the 21st century, the Sol-Gel method has continued to evolve and find applications in diverse fields (see Figure 2). Its versatility has been harnessed in the synthesis of nanomaterials, biomaterials, and advanced functional materials for electronics, catalysis, sensors, energy storage, and more.

### 2.2. Sol-Gel Basic Strategies

The Sol-Gel process involves several basic strategies that are essential for the successful synthesis of materials. These strategies revolve around the manipulation of precursor chemistry, reaction conditions, and post-treatment processes to control the formation of the desired materials. Here are the key basic strategies in the Sol-Gel process:

- Choice of precursors: The first step in the Sol-Gel process is the selection of appropriate precursors. Metal alkoxides are commonly used because of their high reactivity and solubility in organic solvents, but metal chlorides and metal acetylacetonates are also employed. The choice of precursors influences the composition and properties of the resulting material.
  Nucleophilic reactions are central to Sol-Gel chemistry, driving the reactivity of metal alkoxides. The degree of reactivity is chiefly influenced by factors such as the electronegativity of the metal atom, its coordination number, and the steric hindrance posed by alkoxide groups. For example, silicon alkoxides exhibit relatively low reactivity, necessitating the use of acid or base catalysis, or nucleophilic activation to enhance hydrolysis and condensation rates. On the other hand, transition metal alkoxides tend to be overly reactive, necessitating stabilization through complexation to avoid rapid condensation [17].
- Hydrolysis and condensation reactions: The Sol-Gel process involves two main reactions: hydrolysis and condensation. During hydrolysis, the metal alkoxide precursor reacts with water or a hydroxide ion to produce metal hydroxides or metal oxides. The hydrolyzed species then undergo condensation, where they react with each other to form metal–oxygen–metal bonds, leading to the formation of a three-dimensional network structure.
- Controlled stoichiometry: Precise control over the stoichiometry of the precursor components is crucial to achieve the desired composition of the final material. The molar ratios of water, alcohol (solvent), and metal alkoxide must be carefully adjusted to control the hydrolysis and condensation reactions and prevent undesired side reactions.
- Solvent choice: The choice of solvent is critical as it influences the solubility of the precursors, the rate of hydrolysis and condensation reactions, and the stability of the sol. Common solvents include alcohols (methanol, ethanol, isopropanol) and water. The solvent should be compatible with the precursors and provide a homogeneous sol.
- pH control: The pH of the Sol-Gel system plays a vital role in determining the extent of hydrolysis and condensation reactions. Adjusting the pH to a specific range can promote the formation of the desired material and prevent the precipitation of unwanted phases.
- Aging and gelation: permitting the sol to undergo an aging period facilitates additional condensation reactions and structural rearrangement, which culminates in the formation of a cohesive gel network. This aging phase allows the colloidal solution to evolve and stabilize, promoting the development of stronger metal–oxygen–metal linkages within the material. As the aging progresses, the gel structure becomes more pronounced, leading to the critical point of gelation. Gelation marks the transformation of the ‘sol’ from a liquid colloidal state to a solid gel with a continuous three-dimensional framework. At this pivotal moment, the gel firmly retains the liquid phase within its structure, achieving the characteristic properties and structure of the final material.
- Drying and post-treatment: After phase separation of the gel by centrifugation, the gel is typically dried to remove the solvent and produce a porous solid. Depending on the desired application and properties, post-treatment steps such as calcination/sintering or surface modifications may be carried out to enhance the final material’s characteristics.

**Figure 3:**
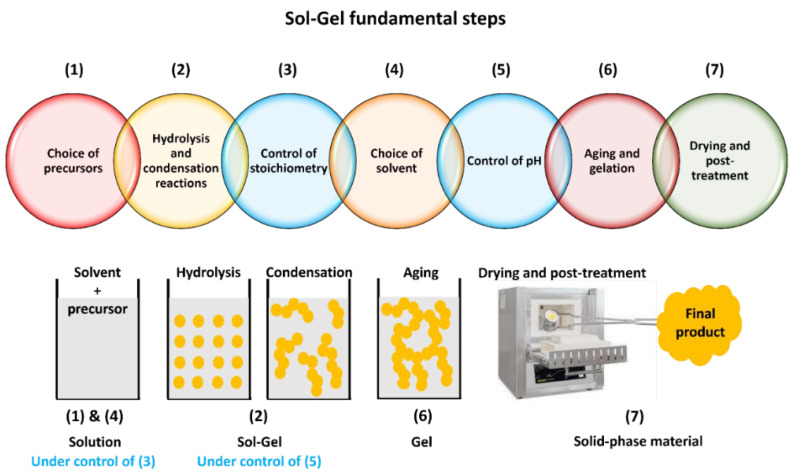
Sketch of Sol-Gel steps.

All these steps are summarized and schematized in Figure 3.

**Figure 4:**
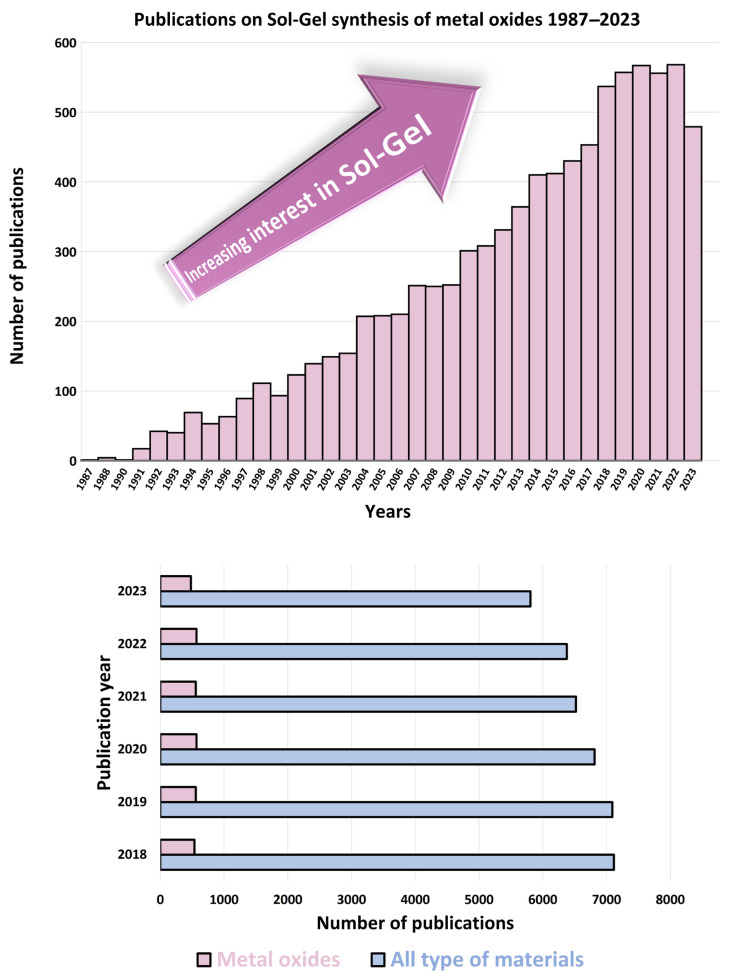
Number of publications on Sol-Gel synthesis of metal oxides (1987–2023) (**Top**) and its comparison with synthesis of other materials for the last five years (**Bottom**), from Web of Science using the search words ‘Sol-Gel’ and ‘metal oxides’, 31 January 2024.

By carefully controlling these fundamental steps, researchers can tailor the composition, structure, and properties of the materials obtained through the Sol-Gel process, making it a versatile and widely used technique in materials synthesis. In Figure 4, we show the increasing interest in recent decades in the Sol-Gel technique to prepare metal oxide materials.

## 3. Sol-Gel-Derived MOX and Associated Morphologies

The Sol-Gel method is a widely used process for producing materials in various forms: porous materials, fibers, nanosized powders, hierarchical morphologies, etc. Different groups have used the Sol-Gel method to produce metal oxide nanostructures, and we outline a selection of these studies in the following.

- **ZnO**

Various morphologies of ZnO are achievable when using the Sol-Gel method. Krasteva et al. [18] prepared an array of nanorods on a ceramic substrate whose diameter and length were 0.5 µm and 13.5 µm, by using 10 g of zinc acetate, 20 g of 2-methoxyethanol, and 3.2 g of 2-aminoethanol, agitated it for 15 min, and then heated it to 60 °C for 1 h. Zinc acetate was also used by Bagheri Khatibani [19] to make ZnO nanoparticles. He poured diethanolamine (DEA) into ethanol, stirring for 10 min. Then, he added the precursor to the solution and stirred it for 2 h until it became clear and homogeneous, followed by an aging process for 24 h at room temperature.

**Figure 5:**
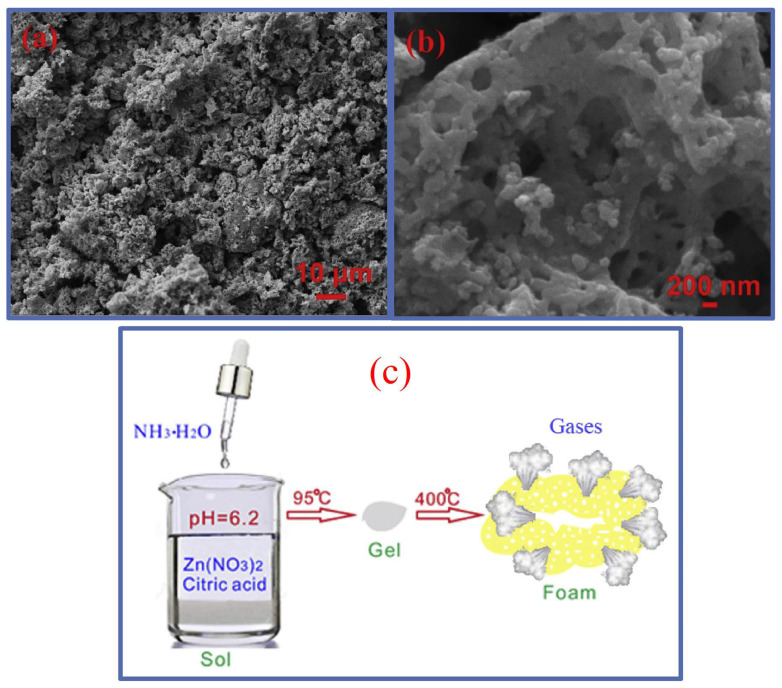
(**a**,**b**) SEM images of Sol-Gel-derived porous structure of zinc oxide at different magnifications, and (**c**) schematic illustration of ZnO synthesis. Reprinted/adapted with permission from Ref. [20]. 2019, Elsevier.

A porous structure could also be prepared [20] by dissolving 5 mmol of zinc nitrate in 30 mL of distilled water, followed by the addition of 1.5 mmol of citric acid under a continuous magnetic stirring of 2500 rpm until adjusting the pH to 6.2 with ammonia dropwise. The colloidal solution was heated at 95 °C for 50 h to evaporate the solvent and form the dry gel, which was heated thereafter at 400 °C for 1 h to obtain the ZnO foam with holes, as shown in Figure 5.

**Figure 6:**
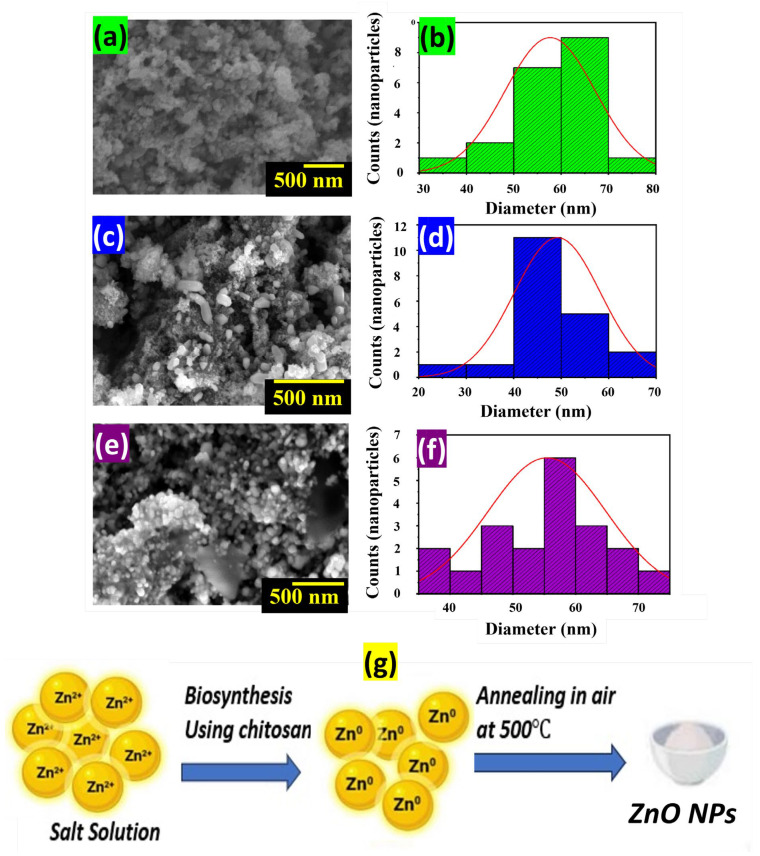
SEM characterization of ZnO nanoparticles using different chitosan sources: (**a**,**b**) crab shells, (**c**,**d**) shrimp shells, and (**e**,**f**) streptomyces griseus bacteria, and (**g**) schematic illustration of the synthesis process. Reprinted/adapted with permission from Ref. [21]. 2022, MDPI.

In another work, chitosan was chosen for its ability to interact with zinc ions through amino and hydroxyl groups to prepare ZnO nanoparticles (NPs). Upon mixing chitosan and zinc chloride (ZnCl_2_), Zn^2+^ ions hydrolyzed, forming Zn(OH)_2_, which then bound to chitosan. The solution stability of chitosan/Zn(OH)_2_ was achieved by raising the solution’s pH to 9 with NaOH. Subsequent drying at 100 °C and annealing at 500 °C in air were applied in order to completely remove chitosan and to crystallize ZnO NPs [21] (see Figure 6).

- **V_2_O_5_**

A free-surfactant synthesis of a V_2_O_5_ porous-flower-like nanostructure was performed by Dhayal Raj et al. [22]. They prepared a clear solution with a 30 mmol concentration by combining 0.05 M of ammonium metavanadate with 1.2 mL of ethylene glycol and stirring at room temperature for 1 h. The solution was then heated to 140 °C, continuously stirred for 4 h, followed by natural cooling to room temperature. The likely chemical reaction is represented as

2NH_4_VO_3_ + 3HOCH_2_CH_2_OH → 2VO(OCH_2_CH_2_O) + HOCH_2_CHO + 4H_2_O + 2NH_3_

**Figure 7:**
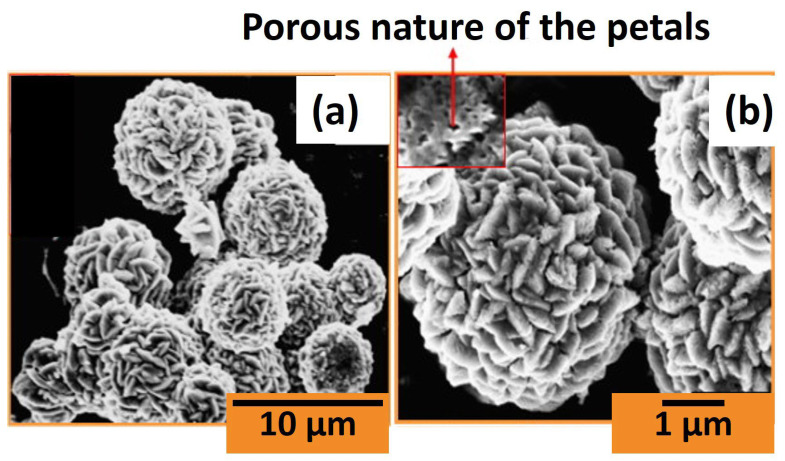
SEM micrographs of V_2_O_5_ stirred for 4 h at (**a**) 140 °C and (**b**) its magnified image, the red box indicates the porosity of petals. Reprinted/adapted with permission from Ref. [22]. 2012, Elsevier.

The general reaction mechanism involves the thermal decomposition of ammonium metavanadate in ethylene glycol, leading to the formation of vanadium oxide clusters. As the transition from a liquid to a solid precipitate takes place, numerous energy-related factors come into play, affecting the formation of larger precipitates resembling expanded plate-like structures through the process of Ostwald ripening. The central area of each petal appears broader, resulting in the accumulation of more particles, gradually tapering towards the edges of the plate. Notably, as the vanadium glycolate chain lengthens, the structure condenses into a closed spherical shape. These condensed vanadium glycolate spheres serve as anchor points for the plate-like structures, culminating in the formation of a flower-like structure with nanopetals, as described in Figure 7.

- **CeO_2_**

**Figure 8:**
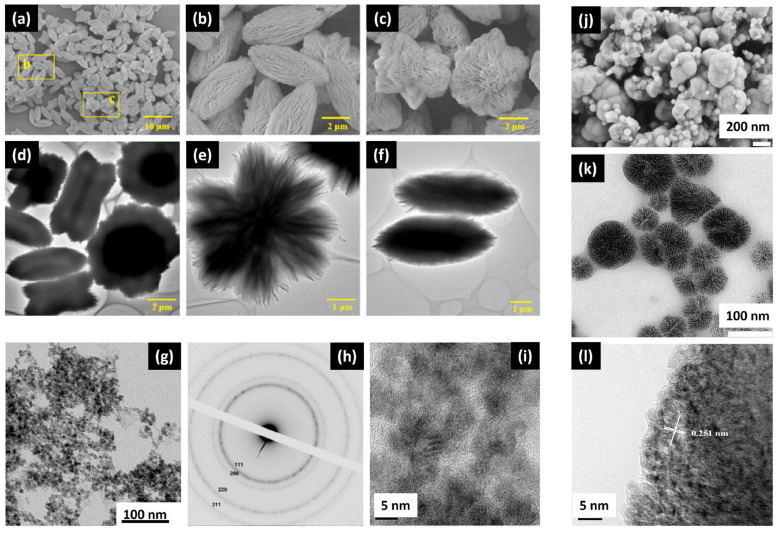
SEM and TEM micrographs of hierarchical ceria nanoparticles using different precursors: (**a**–**f**) cerium oxalate, reprinted/adapted with permission from Ref. [25]. 2015, Elsevier; (**g**–**i**) cerium chloride, reprinted/adapted with permission from Ref. [24]. 2010, Elsevier; and (**j**–**l**) cerium nitrate, reprinted/adapted with permission from Ref. [23]. 2009, ACS.

Hierarchical CeO_2_ nanocrystal microspheres were prepared using different cerium-based precursors, as shown in Figure 8 and described in the following.

An amount of 1 mmol of cerium nitrate Ce(NO_3_)_3_·6H_2_O was dissolved in 10 mL of benzyl alcohol, under stirring for 2 days at 120 °C. The gel was centrifuged, washed with anhydrous ethanol, and dried in air at 60 °C to give CeO_2_ nanocrystal microspheres with hierarchical form [23].

Also, cerium chloride CeCl_3_ can be used to prepare ceria nanoparticles via the Sol-Gel method. Wang et al. [24] mixed it with cetyltrimethylammonium bromide (CTAB) as a cationic surfactant and NH_3_·H_2_O solution at room temperature. After 24 h of stirring, the mixture was left to age at room temperature for 10 days. The resulting product was filtered, rinsed with distilled water to eliminate the surfactant, and then air-dried.

Another research group prepared two alcohol-based solutions containing Ce^3+^ and oxalic acid, mixed at 50 °C while being vigorously stirred, resulting in the rapid formation of a white gel. An additional 0.5 h stirring period, followed by centrifugation, ethanol washing, subsequent drying at 80 °C overnight, and calcination in air at 450 °C for 2 h led to the final hierarchical CeO_2_ powder [25].

**Figure 9:**
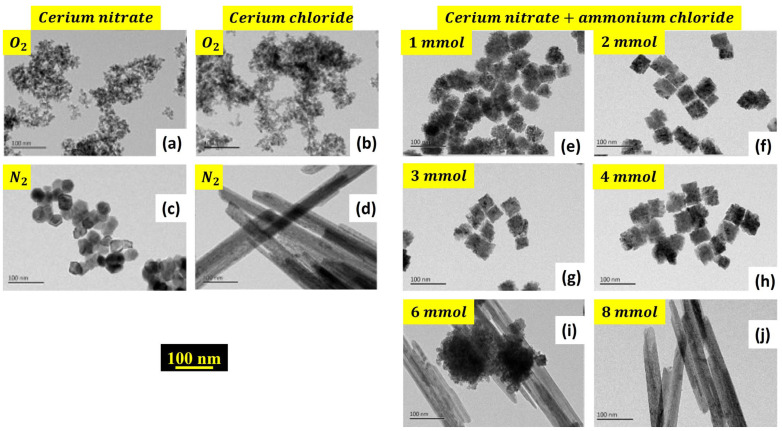
TEM images of ceria nanopowder at the same magnification prepared under oxygen atmosphere (**a**,**b**) and nitrogen atmosphere (**c**,**d**) using two different cerium precursors (cerium nitrate and cerium chloride). (**e**–**j**) TEM images of ceria nanostructures using cerium nitrate as precursor and ammonium chloride as surfactant agent with varying the Cl^–^ amount: (**e**) 1 mmol, (**f**) 2 mmol, (**g**) 3 mmol, (**h**) 4 mmol, (**i**) 6 mmol, (**j**) 8 mmol. Reprinted/adapted with permission from Ref. [26]. 2018, Elsevier.

In an interesting research work, Wang et al. [26] studied the morphology transformation of ceria from nanoparticles to nanorods under an inert atmosphere without any surfactant or template. They prepared different sets using precursors of CeCl_3_·7H_2_O and Ce(NO_3_)_3_·6H_2_O, resulting in various morphologies such as irregular nanoparticles, rectangular polycrystals, truncated octahedral single crystals, and nanorods. The first set was prepared under different atmospheres using a cerium nitrate precursor: the irregular shapes of ceria nanoparticles that were synthesized were strongly agglomerated in N_2_ and O_2_ environments, unlike the particles prepared in air [26]. Even the crystallinity of the ceria powder prepared in air was much higher than that of the powder prepared in other environments, indicating a cubic fluorite structure with an Fm-3m space group (JCPDS 34–0394) (see Figure 9). Comparing cerium-chloride-based powder with cerium-nitrate-based powder, a noticeable shift in the peaks to the left is mentioned, indicating a higher lattice parameter in the cerium-chloride-based powder (5.41892 Å vs. 5.40795 Å) with a lower crystallinity. This expansion is due to the presence of oxygen vacancies and Ce^3+^ ions that alter Ce-O bond lengths (Ce^4+^ → Ce^3+^). The authors [26] reported that chloride ions can significantly affect the shape of CeO_2_ nanoparticles. Adding ammonium chloride delays NO production, indicating a postponement of oxidation. With 1 mmol NH_4_Cl, NO appears after 20 min at 80 °C, but the particles change from single crystals to irregular polycrystals. Using 2 mmol and 3 mmol NH_4_Cl delays NO generation to nearly 30 min, with rectangular polycrystals forming. Higher NH_4_Cl amounts further delay NO production, completely stopping it at 8 mmol and 10 mmol NH_4_Cl, resulting in rod-like CeO_2_. At 6 mmol NH_4_Cl, oxidation is inhibited (uncomplete process), yielding a mix of nanoparticles, nanorods, and rectangular particles.

- **MnO_2_**

**Figure 10:**
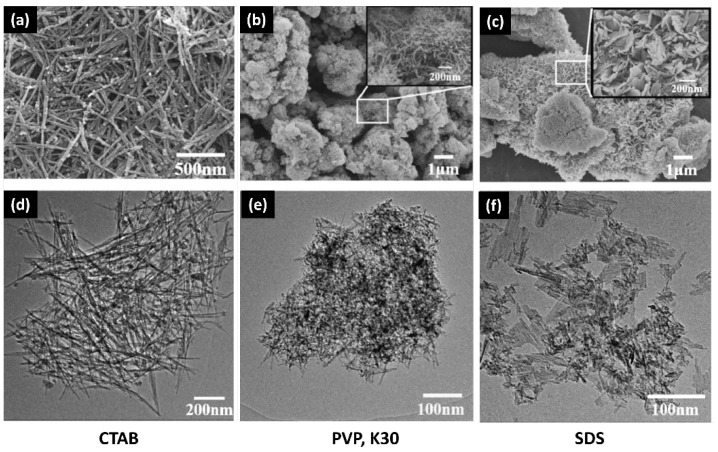
SEM images of MnO_2_ using different surfactant agents: (**a**) CTAB, (**b**) PVP, K30, and (**c**) SDS. The insets in (**b**) and (**c**) are zoom on SEM images of MnO_2_ at 200 nm scale. TEM images of MnO_2_ using PVP (**d**), K30 (**e**) and SDS (**f**) surfactants. Reprinted/adapted with permission from Ref. [27]. 2014, Elsevier.

Using various surfactant agents plays an important role in altering the morphology of Sol-Gel-prepared metal oxides. Tang et al. [27] studied the surfactant’s impact on MnO_2_, as shown in Figure 10. When CTAB is employed as the surfactant, it facilitates the creation of highly dispersed ultrafine nanowires. Conversely, the use of polyvinyl pyrrolidone (PVP, K30) or pluronic triblock copolymer (P123) leads to the synthesis of microparticles aggregated with nanowires. Sodium dodecyl sulfate (SDS), on the other hand, drives the formation of sheet-like structures assembled with nanorods. (see Figure 10).

- **CuO**

**Figure 11:**
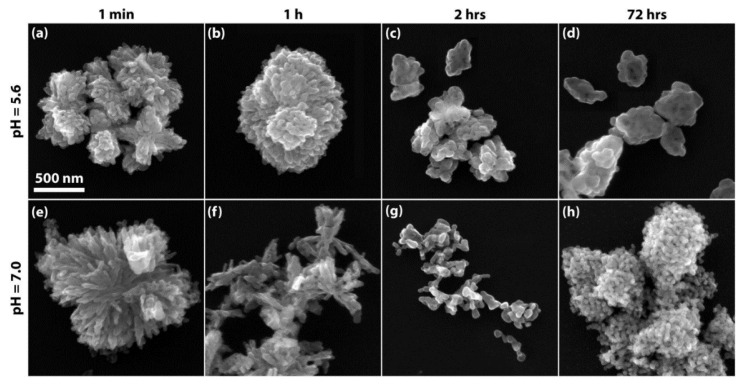
Morphologies of CuO nanopowder synthesized via Sol-Gel at two pH values. At pH = 5.6, the aging times are: (**a**) 1 min, (**b**) 1 h, (**c**) 2 h, (**d**) 72 h. At pH = 7, the aging times are: (**e**) 1 min, (**f**) 1 h, (**g**) 2 h, (**h**) 72 h. Reprinted/adapted with permission from Ref. [28]. 2019, Springer Nature.

Aging time and solution pH are crucial factors in Sol-Gel synthesis. Dörner et al. [28] prepared CuO nanopowder at different pH values and precipitation times, as shown in Figure 11. For the shortest stirring period, the aggregated nanoparticles (NPs) and agglomerates that were formed at both pH levels exhibited needle- and platelet-like crystallites. For the longest stirring time, the aggregation of nanoparticles was the highest, where CuO aggregates were densely clustered for both pH levels. However, for a stirring time of 2 h, the clustering density of CuO NPs was much lower, resulting in more nanoporous powder. It is worth noting that the agglomeration effect at low pH levels was more pronounced where the structure was more compacted. The authors attributed this finding to the [CO_3_^2−^]/[Cu^2+^] molar ratio (0.4 at pH = 5.6 vs. 2.5 at pH = 2.5): the compactness is higher for a carbonate-deficient solution (pH = 5.6) where neighboring Cu cores in the precipitated gel are much closer to each other, resulting in denser clustering. Notably, TEM analysis confirmed that the primary crystallites prepared at high pH levels exhibited a more spherical and smoother shape, and the resulting CuO aggregates appeared less densely packed with an average size of 38 nm, larger than that obtained at low pH levels (23 nm). This could be explained by the fact that carbonate molarity affects the solubility of the solid precursor within the mixed solution and its composition, which in turn influences the final size resulting from Oswald ripening during the stirring process.

- **NiO**

**Figure 12:**
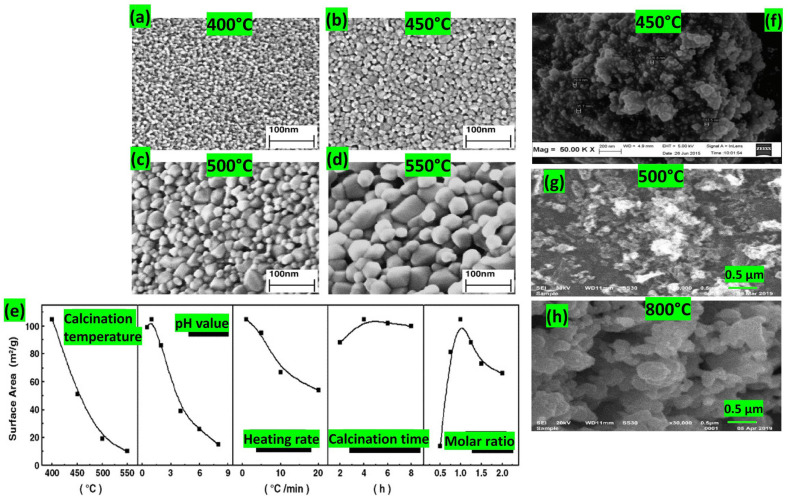
(**a**–**d**) SEM images showing the effect of calcination temperature on NiO morphology, (**e**) surface area dependency on calcination temperature, pH value, heating rate, calcination time, and molar ratio, reprinted/adapted with permission from Ref. [31]. 2007, Elsevier, SEM image of NiO calcined at (**f**) 450 °C, reprinted/adapted with permission from Ref. [29]. 2016, Elsevier, and (**g**) 500 °C, and (**h**) 800 °C , reprinted/adapted with permission from Ref. [32]. 2020, AIP Publishing.

Zorkipli and colleagues prepared NiO nanoparticles without impurities with an average diameter of 32.9 nm by dissolving nickel (II) nitrate hexahydrate (Ni(NO_3_)_2_·6H_2_O in 20 mL of isopropanol alcohol and 20 mL of polyethylene glycol and stirring the solution continuously for 24 h until complete dissolution was achieved. Ammonium hydroxide (NH_4_OH) was subsequently added to adjust the pH of the mixture to 11. To prevent particle agglomeration, triton X-100 was introduced. The solutions were then heated to 80 °C, leading to the formation of the gel, which was subjected to drying at 200 °C and calcining at 450 °C [29]. Calcination temperature can influence metal oxide nanoparticles as reported by Kavitha et al. [30]: the NiO nanoparticles displayed a uniform distribution and adhered to each other, creating agglomerated particles. As the annealing temperature was raised from 500 to 700 °C, the aggregate size grew, causing an augmentation in the crystallite size of the particles. Wu et al. [31] also studied the calcination parameters, proving that (i) lower temperatures provide a uniform distribution and small size of nanoparticles, (ii) higher heating rates increase the particle size and drastically reduce the surface area (1 °C/min is preferable for NiO), and (iii) shorter calcination times can prohibit the total decomposition of precursors, leading to low surface areas and larger nanoparticles. Moreover, they highlighted that the optimal preparation of NiO nanoparticles with uniformity and minimal agglomeration requires the molar mass ratio of citric acid to nickel nitrate to be maintained at an equivalence. The pH value of the solution should be controlled to remain relatively acidic with the optimal value being 1. Additionally, it is essential to minimize the calcination temperature and duration while ensuring complete decomposition of the precursor (see Figure 12).

- **Co_3_O_4_**

Cobalt oxide nanorods were prepared using 5 g of cobalt nitrate precursor dissolved in 100 mL of de-ionized water under stirring, followed by slowly adding 100 mL of ethanol and stirring for 5 min at room temperature. A CTAB surfactant agent was added to the solution, maintaining it at pH = 5. The gel was dried at 80 °C for 2 h, cooled to room temperature, and then calcined at 600 °C for 3 h without any washing or purification steps, resulting in Co_3_O_4_ nanopowder [33]. After calcination, the nanoparticle diameter increased from 15–30 nm to 50 nm. This was explained by the fact that removing the CTAB at 600 °C allows the particles to be closer to each other.

**Figure 13:**
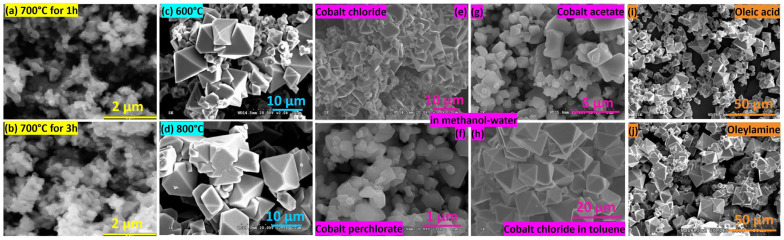
Sol-Gel preparation of cobalt oxide under different conditions: (**a**,**b**) effect of calcination time on the morphology. Reprinted/adapted with permission from Ref. [34]. 2020, AIP Publishing. (**c**,**d**) effect of calcination temperature, (**e**–**h**) effect of metal precursor and solvent, and (**i**,**j**) effect of capping agents added to cobalt (II)-chloride-hexahydrate-based solution on nanopowder morphology. Reprinted/adapted with permission from Ref. [35]. 2019, Elsevier.

Sardjono et al. [34] combined cobalt (II) nitrate with ethylene glycol in a 1:3 ratio. They then stirred the mixture at 350 rpm using a magnetic stirrer for 2 h at room temperature to ensure a homogeneous solution. Subsequently, they increased the temperature to 90 °C and continued stirring the solution at the same speed until it was transformed into a gel-like substance. After drying the cobalt oxide gel at 120 °C, they calcined it at 700 °C at different holding times. The longest calcination time (3 h) showed the smallest particles when compared to other particles annealed for 1 h and 2 h. However, the 2 h calcined powder had the smallest crystallite size with a more agglomerated structure due to the magnetic properties of the cobalt oxide. Increasing the calcination temperature can induce larger particles and increase the crystallite size by providing the activation energy necessary for increasing the growth rate [35]. In Figure 13c,d, a clear octahedral geometry is observed at low temperature (600 °C) with a non-uniform distribution of nanoparticles. However, heating to 800 °C during calcination resulted in a combination of octahedral and truncated octahedral shapes, accompanied by particle sintering. This occurrence was explained by the melting of the more delicate facets, corners, and edges of the particles at elevated calcination temperatures [35].

The effects of solvents, metal precursors, and capping agents on the cobalt oxide structure were studied by Itteboina et al. [35] (see Figure 13e–j). When dissolving cobalt chloride hexahydrate in two different solvents, the particles prepared with methanol–water tended to aggregate in the presence of prism and hexagonal shapes. Conversely, particles synthesized in toluene exhibited a uniform size, smooth surfaces, and well-defined sharp edges and corners with larger sizes. The difference in size and morphology was explained by the binding of the organic species to the particle surface during the nucleation in polar/non-polar reaction environment.

Capping agents such as oleic acid and oleylamine have a great impact on cobalt oxide particles prepared in methanol–water environments, as can be seen in Figure 13i,j. Octahedral particles utilizing oleic acid demonstrate a smaller size compared to those using oleylamine. This disparity arises from the ionic behavior of oleic acid, which exhibits a strong capping strength, effectively reducing the agglomeration effect [35].

## 4. Gas Sensing Application

### 4.1. MOX Gas Sensing Mechanismn

**Figure 14:**
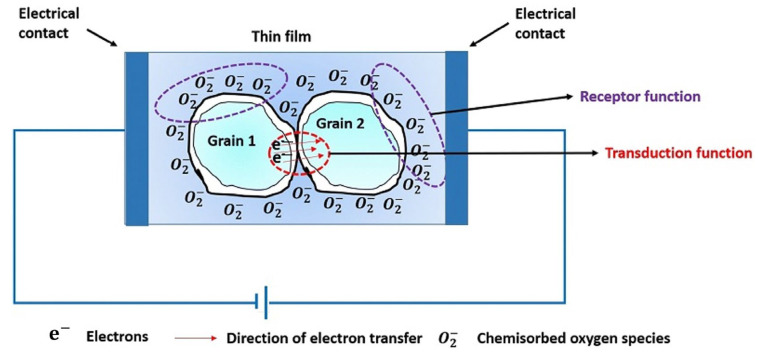
Schematic of MOX thin-film gas sensor mechanisms. Reprinted/adapted with permission from Ref. [36]. 2019, Springer Nature.

It is essential to uncover the sensing mechanism used by metal oxide gas sensors, as this knowledge is useful for preparing novel gas sensing materials that exhibit superior performance. While the precise underlying mechanisms responsible for the gas response remain a topic of debate, they mainly involve changes in conductivity resulting from the trapping of electrons in molecules adsorbed on the sensor surface, along with changes in band structure caused by these charged molecules. The gas molecules can serve as charge carriers (acting as donors or acceptors) in a receptor capacity, which in turn modifies the resistivity of the metal oxide in a transduction role, as illustrated in Figure 14.

The variation in resistance of the MOX thin film depends on both the dominant carriers in the semiconductor film and the specific gas molecules present in the surrounding atmosphere, whether they possess oxidizing or reducing properties. In the case of n-type materials, oxidizing gases (called acceptors) cause an increase in the resistance of the thin film, while reducing gases (called donors) cause a decrease. Conversely, for p-type materials, the effects are reversed [37].

In practice, for example, for an n-type MOX, oxygen gas adheres to the surface of the MOX sensing material when exposed to air. These oxygen species that attach to the surface can capture electrons inside the MOX film. The accumulation of negative charges within these oxygen species results in the formation of a depletion layer, leading to a decrease in conductivity. When the reducing gas encounters the sensing surface, the electrons trapped by the oxygen adsorbate are released into the MOX film. This clearance causes a reduction in the height of the potential barrier, therefore increasing conductivity. It is important to note that there are different forms of oxygen species, including molecular (O_2_^−^) and atomic (O^−^, O^2−^) ions on the surface, depending on the working temperature. Generally, at temperatures below 150 °C, the molecular form prevails, while at temperatures above 150 °C, the atomic species become more important [38,39,40,41]. As the temperature increases, a chemical reaction occurs on the MOX films, where (O_2_^−^_ads_ + e^−^) transforms into 2O^−^_ads_. The desorption temperatures of O^−^_ads_ ions are approximately 550 °C, while for O_2_^−^_ads_ ions, they are approximately 150 °C when released from the MOX surface. Under constant oxygen coverage, this transition results in an increase in surface charge density, causing variations in band bending and surface conductivity.

### 4.2. Sensing Performance

- **Gas response**

**Figure 15:**
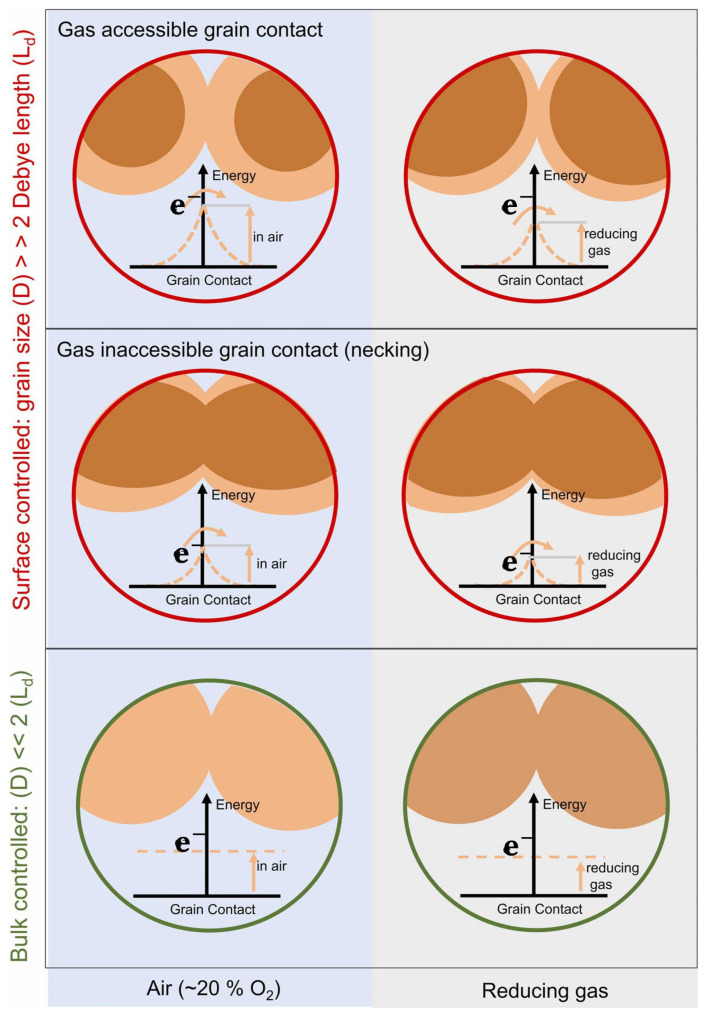
Three mechanisms of grain size dependence of conductance in semiconductor gas sensing materials: D ≫ 2 L grain boundary control, D = 2 L neck control, D < 2 L grain control. Reprinted/adapted with permission from Ref. [43]. 2022, Elsevier.

The sensor’s response can be greatly enhanced through alterations in its microstructure, such as adjusting the grain size. Figure 15 depicts the three mechanisms that govern the relationship between grain size and conductance in semiconductor gas sensing materials. In the case of large grains, where the grain size (D) is much greater than twice the thickness of the space charge layer (2 L), the conductance is controlled by the Schottky barrier present at the grain boundaries, a phenomenon referred to as ‘grain boundary control’. When D equals 2 L, conductance is constrained by the narrow connections between grains, referred to as ‘neck control.’ On the other hand, when D is less than 2 L, conductance is governed by each individual grain, known as ‘grain control’ [42,43].

An alternative approach to augment sensitivity involves altering the porosity of MOXs. A greater porosity, which results in larger surface areas, has been observed to heighten gas sensitivity. The gas response of MOX sensors can also be improved by introducing dopants or impurities, such as noble metals like Pt [44], Nb [45], metal oxide PdO [46], and rare earth oxide CeO_2_ [47]. It is noteworthy that environmental humidity significantly influences the performance of MOX gas sensors. Increased humidity levels have been linked to a reduction in the response of metal oxide sensors, as demonstrated in reference [48].

- **Selectivity**

Enhancing the selectivity of semiconductor metal oxide gas sensors poses one of the key challenges. There are typically two approaches available to improve the selectivity of MOX gas sensors. The first one is to create a material that exhibits selectivity to a specific compound while maintaining a minimal or zero sensing response to other substances that might be present in the same working environment. The other method involves distinguishing among multiple substances within the mixture, typically accomplished by either adjusting the temperature [49] or by using sensor arrays [49,50]. Also, the introduction of dopants or impurities into metal oxides, or the creation of mixed metal oxides, can similarly boost the gas sensors’ selectivity. This occurs because each material exhibits specific response spectra to gas species.

- **Stability**

Stability stands as a crucial factor in the creation of gas sensors for practical use, as these sensors are expected to generate a consistent and replicable signal, ideally for a duration of 2 to 3 years, equivalent to approximately 17,000 to 26,000 h of continuous operation [51]. Sensor stability can be categorized into two types. The first type pertains to the sensor’s ability to maintain consistent characteristics over a specified period while operating under challenging conditions, such as high temperatures and exposure to a known analyte. This type of stability is commonly referred to as ‘active stability’. The second type of stability is associated with the sensor’s capacity to preserve its sensitivity and selectivity over time under standard storage conditions, like room temperature and ambient humidity. This is often known as ‘passive stability’.

Possible causes of instability could include structural changes, phase transitions, contamination, contact and heater deterioration, bulk diffusion, design errors, variations in humidity, temperature fluctuations in the surrounding environment, and interference effects [51]. Enhancing stability can be achieved by employing post-processing methods such as calcination and annealing, as well as by lowering the operational temperature of the sensing component. Furthermore, augmenting sensor element stability is attainable through techniques like doping metal oxides with other metals or synthesizing mixed oxides, as mentioned in reference [49].

### 4.3. Characterization Methods

There are three prevalent techniques for characterizing MOX-based gas sensors, resistive, impedimetric, and capacitive analyses, where the electrical properties of gas sensing materials are influenced by a range of factors, including bulk resistance, surface effects, grain boundaries, and the contact between the grain interface and the electrode. Therefore, it is possible to derive a corresponding equivalent circuit diagram [52].

From an electronics standpoint, this component can be characterized as a constant-phase element, and Equation (1) displays its impedance expression.

(1) $$Z=A\times \left[\cos\left(\frac{\pi }{2}\times n\right)-j\times \sin\left(\frac{\pi }{2}\times n\right)\times \omega^{-n}\right]\;\left(0\leq n\leq 1\right)$$

where n takes values of 0, 0.5, and 1, with the corresponding Z values of pure resistance, Warburg impedance, and pure capacitance [53].

### 4.4. N-Type Metal Oxides

- **ZnO**

**Figure 16:**
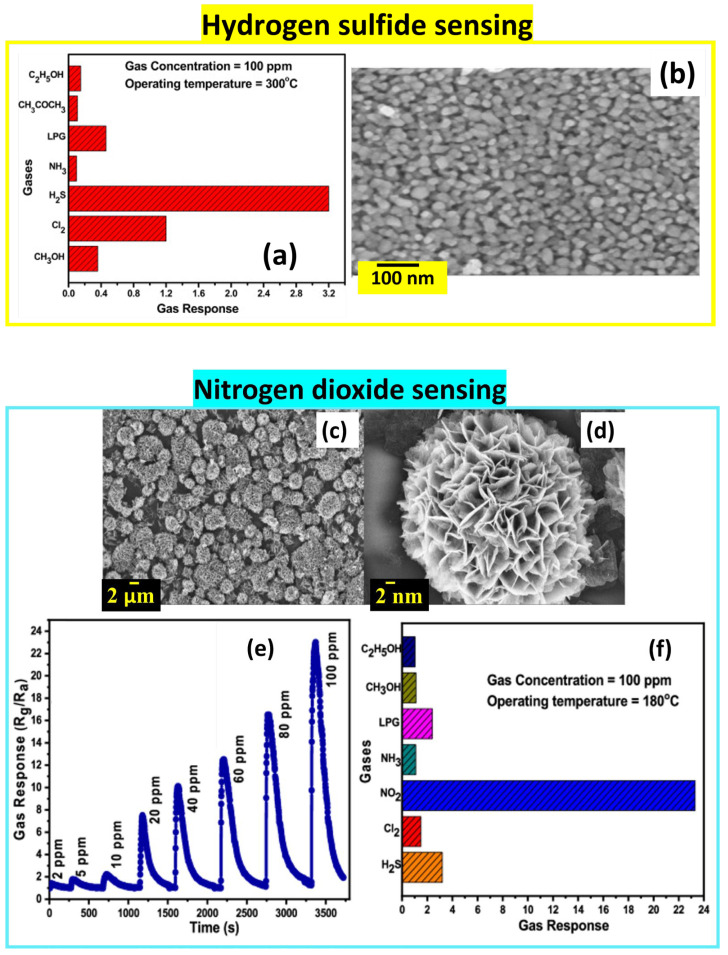
Zinc oxide as gas sensor to H_2_S: (**a**) Selectivity measurements to different gases at 300 °C and (**b**) SEM image of ZnO nanoparticles. Reprinted/adapted with permission from Ref. [54]. 2017, Elsevier. ZnO as NO_2_ sensor: SEM images of ZnO like-flower at (**c**) 2 µm and (**d**) 200 nm, (**e**) dynamic response to NO_2_ at different gas concentrations, (**f**) selectivity measurement at 180°C to different gases. Reprinted/adapted with permission from Ref. [55]. 2021, Springer Nature.

ZnO Sol-Gel-derived nanostructures show high selectivity to hydrogen sulfide among seven reducing gases at 300 °C, a stability of 59%, and a fast response/recovery time (10 s/198 s for 100 ppm). ZnO was prepared by dissolving zinc acetate dihydrate in ethanol and m-cresol and stirring the mixture at 70 °C for 90 min. The homogeneous solution was spin-coated on the glass substrate at 2000 rpm for 30 s and dried at 200 °C for 5 min. After repeating the deposition 10 times, the sample was annealed at 600 °C for 1 h, showing a nanocrystalline film with an average particle size around 30.5 nm, as seen in Figure 16b [54].

ZnO is also presented herein as a NO_2_ gas sensor [55] working at an optimized temperature of 180 °C that provides the necessary activation energy for complete chemical reactions on the sensing layer with a fast response time (4 s for 10 ppm and 14 s for 100 ppm) and stability of 69% after 40 days up to 90 days.

ZnO sensing films were prepared by using the same precursor and solvent mentioned above, stirred at 70 °C for 120 min. Then, four drops of the solution were cast on the glass substrate and annealed in air after three days at 500 °C for 2 h, leading to a 3D flower-like structure with porous petals that justify the high sensing performance of the ZnO sensor [55] (see Figure 16c–f).

- **SnO_2_**

Several groups reported the formation of SnO_2_ nanostructures via Sol-Gel for sensing applications. Beniwal et al. dissolved 2 g of tin chloride and 2.5 mL of acetic acid in 100 mL of methanol. Subsequently, the solution was stirred for 50 min under ambient conditions until the precursor mixture achieved complete homogeneity. Additionally, glycerin was introduced at a volume ratio of 2:10 for better quality while maintaining stirring under ambient conditions for 3 h followed by heating at 75 °C for 8 h. The sol was aged for 36 h at room temperature. Then, the SnO_2_ film was spin-coated on the interdigitated-contact-patterned alumina substrate at a speed of 3200 rpm for 30 s and heated at 90 °C for 12 min to dry and remove the organic solvent. Afterwards, a 2 h annealing of the film was carried out in air at 550 °C to ensure SnO_2_ crystallization, resulting in a flake-like morphology with non-uniform and slackly packed porous grains. This tin dioxide sensor showed a high selectivity to ammonia at room temperature, a low limit of detection (<0.5 ppm), a rapid response/recovery time of 16 s/18 s, repeatability, and high reproducibility at extremely low and high concentrations of ammonia [56].

**Figure 17:**
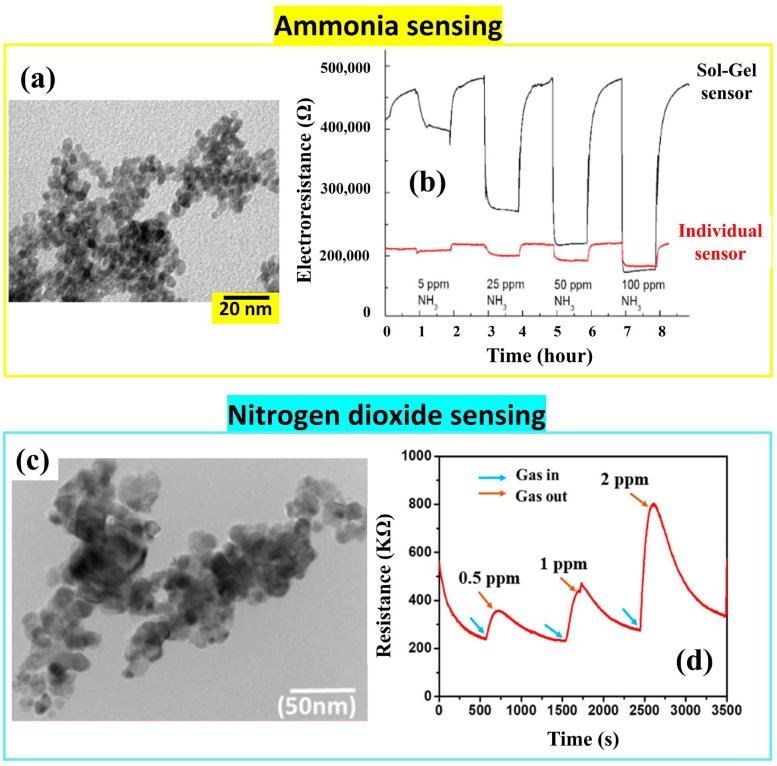
Sensing properties of SnO_2_ to ammonia (**a**) TEM image of SnO_2_ particles and (**b**) dynamic response to NH_3_ of Sol-Gel derived sensor and individual nanowire sensor. Reprinted/adapted with permission from Ref. [57]. 2019, Beilstein-Institut. SnO_2_ as NO_2_ sensor: (**c**) TEM image of SnO_2_ particles and (**d**) their dynamic response to NO_2_, reprinted/adapted with permission from Ref. [58]. 2019, MDPI.

Another Sol-Gel-derived ammonia sensor is shown in Figure 17, operating at 300 °C [57]. The nanopowder’s morphology seems to be an agglomeration of nanoparticles with a size of 4–6 nm. The authors used tin acetate as a precursor and glacial acetic acid as a solvent. They added hydrogen peroxide to the solution and then aqueous ammonia dropwise to cause hydrolytic precipitation of tin oxide. The colloid was precipitated, dried, and annealed, resulting in a SnO_2_ nanopowder.

At room temperature, tin dioxide can also detect NO_2_ gas as shown in Figure 17d. In order to prepare SnO_2_ nanostructures, Kumar et al. [58] dissolved 2 g of tin chloride dehydrate in 100 mL of distilled water under continuous stirring at room temperature. Then, they added ammonia dropwise while stirring. The colloid was precipitated, centrifuged, and dried at 80 °C for 35 h to remove water molecules and other impurities. The resulting precipitate was annealed at 500 °C for 3 h, giving the SnO_2_ nanoparticles that were dispersed in ethanol and spin-coated on the glass substrate. The spin-coated sensing layer manifested its high selectivity in detecting very low concentrations of NO_2_ with relatively fast response/recovery times (84 s/320 s for 0.5 ppm).

- **WO_3_**

**Figure 18:**
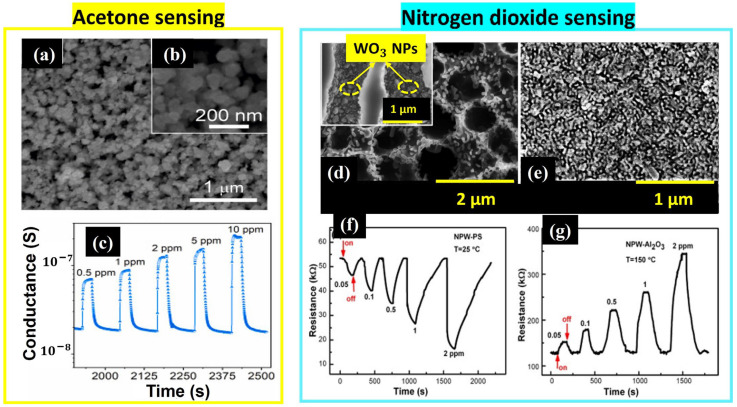
Tungsten trioxide for acetone detection: (**a**) SEM image of WO_3_ nanoparticles, (**b**) inset: SEM image at 200 nm scale, (**c**) dynamic response to different concentrations of acetone. Reprinted/adapted with permission from Ref. [59]. 2023, Elsevier. Tungsten trioxide as NO_2_ sensor: (**d**) SEM image of WO_3_ nanoparticles on porous silicon substrate, inset: zoom on the SEM image at 1 µm scale (**e**) SEM image of WO_3_ nanoparticles on alumina substrate, (**f**) dynamic response to different gas concentrations of WO_3_ on silicon at room temperature, (**g**) dynamic response to different gas concentrations of WO_3_ on alumina at 150 °C. Reprinted/adapted with permission from Ref. [60]. 2014, Elsevier.

Tungsten trioxide nanopowder proved its sensitivity at 400 °C to low concentrations of acetone (from 0.2 to 10 ppm) with high selectivity and good reproducibility [59]. Indeed, Pakdel et al. dissolved 1 g of WCl_6_ in 50 mL of distilled water under stirring for 30 min at room temperature. Then, they added an aqueous solution of vitamin C dropwise until pH = 2 with a reaction time of 5 h. The solution was kept at room temperature for 2 h to settle out before being centrifuged, washed with H_2_O and ethanol, dried at room temperature for 24 h, and annealed at 450 °C for 2 h [59]. The final powder presented a nanoparticle morphology, as one can see in Figure 18a,b.

As highly selective NO_2_ sensors, Yan et al. [60] deposited Sol-Gel-derived WO_3_ powder on two different substrates via dip coating: porous silicon and alumina. This powder was prepared by dissolving 0.8 g of P123 in 10 g of ethanol under stirring until achieving a transparent solution to which 1 g of WCl_6_ was added and stirred for 1 h. The dip-coating was performed at a speed of 4 mm/s followed by drying the samples at 80 °C for 6 h and annealing them at 750 °C for 3 h (2 °C/min) in an argon atmosphere to remove the P123 solvent. WO_3_ deposited on n-type alumina provided a large surface area with an average nanoparticle diameter of 33.3 nm. However, the WO_3_ nanoparticles deposited on p-type porous silicon with a symmetrical hole-shaped frame had higher diameters ranging between 240 and 280 nm, as Figure 18d displays. Both sensors showed a high sensitivity to low NO_2_ concentrations at room temperature. It is worth noting that the nanoparticles deposited on alumina could reach the same response as those deposited on porous silicon when increasing the operating temperature to 150 °C in the range of 0.05–1 ppm. The authors attributed this preference in gas sensing to the p-n heterojunction between porous silicon and WO_3_ nanoparticles and the high porosity (39~40%) of the Si layer with pores of 1.2 μm in diameter and 10 μm in depth that increased the surface-to-volume ratio [59,60].

- **TiO_2_**

**Figure 19:**
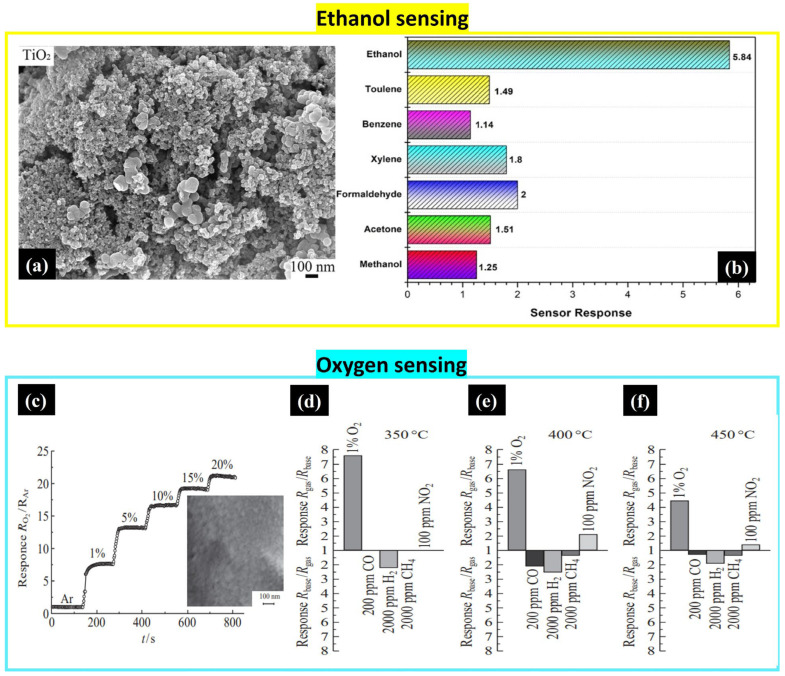
TiO_2_ as ethanol sensor: (**a**) SEM image of TiO_2_ nanoparticles and (**b**) selectivity measurement to different gases. Reprinted/adapted with permission from Ref. [61]. 2023, Elsevier. TiO_2_ as oxygen sensor: (**c**) response to different concentrations of oxygen (inset: SEM image of TiO_2_ nanoparticles at 100 nm scale), selectivity measurements to different gases at (**d**) 350 °C, (**e**) 400 °C, and (**f**) 450 °C. Reprinted/adapted with permission from Ref. [62]. 2018, Elsevier.

TiO_2_ film deposited on a glass substrate via spin coating showed a high selectivity to ethanol as shown in Figure 19. The solution was prepared by adding titanium isopropoxide to an ethanol and diethylene glycol mixture under stirring. Then, it was maintained at room temperature for 30 min. The TiO_2_ nanoparticles with a porous structure could detect 100 ppm of ethanol at room temperature with a response/recovery time of 120 s/156 s for 500 ppm [61].

TiO_2_ also showed its sensitivity to oxygen as mentioned in Sevastyanov et al.’s work. The authors mixed acetylacetone with titanium tetrabutoxide and agitated the solution for 10 min to reduce the hydrolysis reactions until reaching transparent gel. Then, they enabled the hydrolysis activity by adding a solution of water and ethanol (1:1) and agitating it for 2 min. The nanostructured TiO_2_ film was deposited on the alumina substrate via dip-coating at a withdrawal rate of 0.5 mm/min followed by drying for 24 h and annealing in air at 500 °C for 1 h to obtain the crystallized structure. The sensor was tested in different gas environments, showing a high response and high selectivity to O_2_ at 350 °C, which is attributed to the high dispersity of the particles. As can be seen in Figure 19d–f, the sensitivity slightly decreases when increasing the temperature to 450 °C [62].

- **CeO_2_**

**Figure 20:**
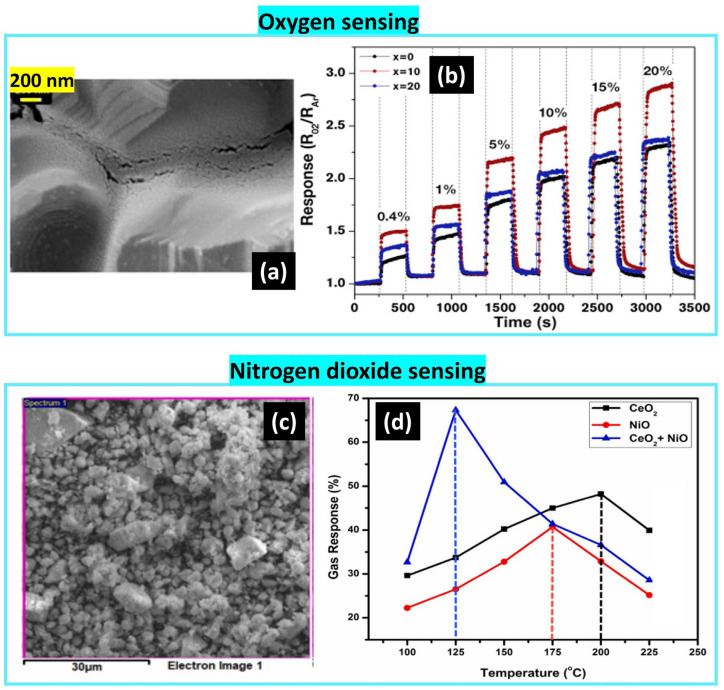
Sol-Gel-derived ceria gas sensor of O_2_: (**a**) SEM image of ceria, and (**b**) dynamic response of ceria (x = 0) and xZrO_2_-ceria (x = 10, 20) to different concentrations. Reprinted/adapted with permission from Ref. [63]. 2019, Elsevier. Ceria as NO_2_ sensor: (**c**) SEM image of ceria particles, and (**d**) gas response of ceria, NiO, and their composite to 25 ppm of NO_2_ at different operating temperatures. Reprinted/adapted with permission from Ref. [64]. 2022, Elsevier.

Mokrushin et al. [63] prepared ceria thin film with an average particle size of 40–55 nm using cerium nitrate and aqueous ammonia. Its deposition was made on polycrystalline alumina with a roughness of 0.4 µm via dip-coating with an extraction rate of 1 mm/s. The sample showed the highest response to oxygen at 400 °C, noting that the sensing performance decreased with humidity (see Figure 20).

In another work, a mixture of cerium nitrate and propylene glycol at a 1:1 ratio was stirred continuously with the addition of aqueous ammonia dropwise until pH = 10. The colloid was washed by distilled water and set in a microwave oven with an on–off cycle for 30 min at 600 W. The cerium oxide powder was ground, resulting in spherical particles with an average size in the range of 25–30 nm and a homogeneous distribution. The sensor was operating at 200 °C as the optimized temperature, showing sensitivity to 10 ppm of NO_2_. Combining it with NiO could reduce the working temperature to 125 °C and significantly increase the response due to the formation of the n-p heterojunction, as shown in Figure 20 [64].

- **V_2_O_5_**

Vanadium oxide can have many crystalline phases such as VO, V_2_O_3_, and V_2_O_4_, which are more stable than the V_2_O_5_ structure. However, vanadium pentoxide has shown interesting properties as a transition metal oxide for gas sensing. It demonstrates its sensitivity to several gases such as helium, acetone, ethanol, hydrogen, ammonia, nitrogen dioxide, and trimethylamine [65,66].

**Figure 21:**
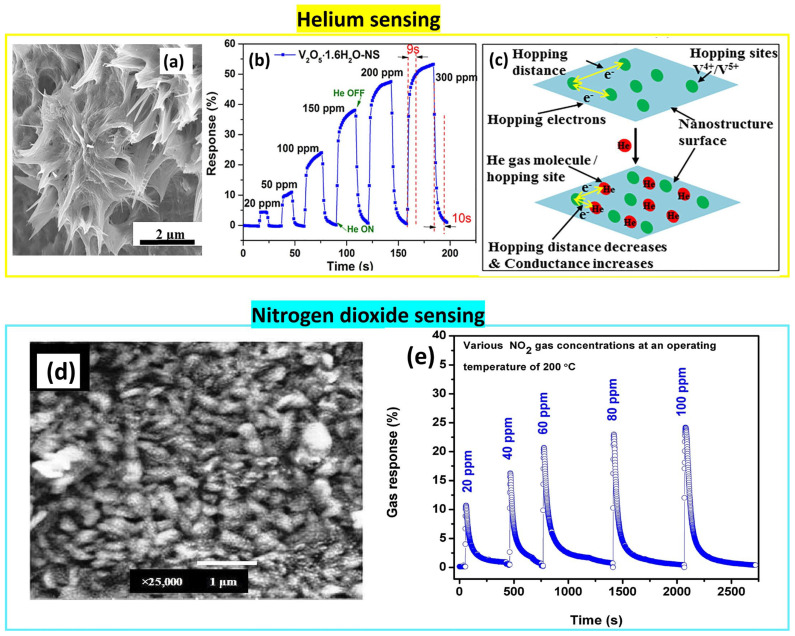
Vanadium pentoxide as He sensor: (**a**) SEM image of V_2_O_5_, (**b**) dynamic response to different concentrations of He and (**c**) schematic illustration of hopping process before and after gas exposure. Reprinted/adapted with permission from Ref. [66]. 2018, Elsevier. V_2_O_5_ as NO_2_ sensor: (**d**) SEM image of V_2_O_5_, and (**e**) dynamic response to different concentrations of NO_2_ at 200 °C. Reprinted/adapted with permission from Ref. [67]. 2017, Elsevier.

V_2_O_5_ is the only oxide that can detect helium, as an inert gas, electrically via the hopping process between V_2_(+IV)O_4_ and V_2_(+V)O_5_, as can be seen in Figure 21. The helium molecules will reduce the distance between the hopping sites, resulting in the increase in the conductance of V_2_O_5_. Chauhan and Bhattacharya prepared V_2_O_5_ nanostructures using the Sol-Gel method. Initially, they dissolved 10 mmol of VOSO_4_ in 40 mL of distilled water, along with 5 mmol of KBrO_3_ under stirring. The resulting solution was stirred for 30 min to ensure homogeneity while maintaining a pH of 1 through nitric acid addition, and then heated at 98 °C for 8 h. After adding 100 mL of deionized water under stirring for 30 min, the solution was heated to 98 °C for 16 h. Subsequently, the resulting product was dried at 80 °C for 12 h, giving V_2_O_5_ nanostars [66]. This nanostructure showed a high selectivity to helium gas compared to other gases (O_2_,CO) with a fast response/recovery time (9 s/10 s) for 300 ppm of He. By contrast, the V_2_O_5_ nanowires exhibited a lower response under exposure to the same gases by demonstrating selectivity, a fast response/recovery time (7 s/9 s), reliability under humid conditions, and stability while detecting helium gas at room temperature [66].

Figure 21 also illustrates the V_2_O_5_ sensitivity to the oxidizing gas NO_2_ at 200 °C with high selectivity [67]. A solution of VCl_3_ with a concentration of 30 mM was prepared by dissolving 0.24 g of VCl_3_ powder in 50 mL of double-distilled water at room temperature and then spray-depositing onto a preheated glass substrate at an optimized substrate temperature of 400 °C to create a V_2_O_5_ sensor. The distance from the spray nozzle to the glass substrate of 27.5 cm and the spray rate of 1.5 mL/min were maintained throughout the deposition process. The sensing layer exhibited a nanorod-like morphology which was responsible for enhancing the sensing performance by enlarging the surface area-to-volume ratio and increasing the pore density, which offered more channels for the diffusion of NO_2_ gas molecules and facilitated electron transport into the sensing layer [67].

### 4.5. P-Type Metal Oxides

- **NiO**

**Figure 22:**
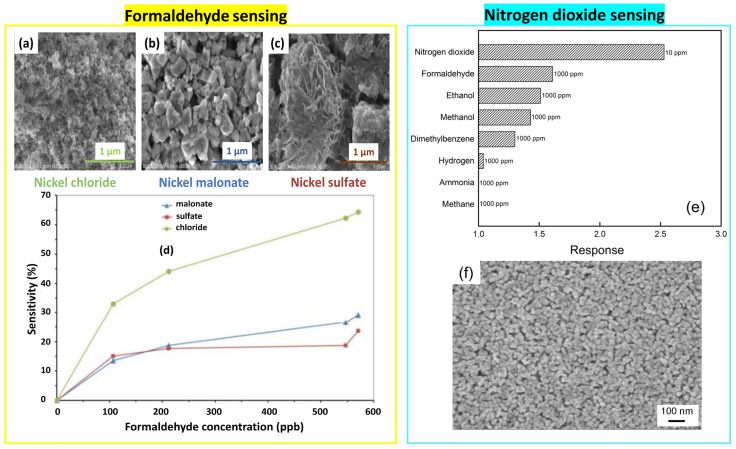
NiO sensitivity to formaldehyde: SEM images of NiO using different precursors (**a**) nickel chloride, (**b**) nickel malonate, and (**c**) nickel sulfate, (**d**) formaldehyde sensitivity of the three sensors at different concentrations. Reprinted/adapted with permission from Ref. [68]. 2016, IOP Publishing. NiO for NO_2_ detection: (**e**) Selectivity measurement and (**f**) SEM image of NiO nanoparticles. Reprinted/adapted with permission from Ref. [69]. 2018, Elsevier.

Nickel oxide powders were synthesized using different precursors for formaldehyde detection [68] (see Figure 22).

(i) An amount of 1.5 g of nickel chloride was dissolved in 70 mL of ethanol at room temperature. NaOH solution was added dropwise to the previous solution and stirred for 2 h to form a light green gel which was washed with distilled water and ethanol after 2 h, filtered, and dried, resulting in small particles uniformly distributed on the surface with an average size of 15–20 nm.
(ii) An amount of 5 mmol of malonic acid was added to 10 mmol of lithium hydroxide to form lithium malonate with pH = 7. After 5 min, the nickel chloride solution was poured into the prepared solution dropwise and continuously stirred. While the solution became green, it was stirred at 90 °C for 3 h. The precipitate was washed successively with distilled water, isopropanol, and acetone, and then dried at 70–80 °C and calcined at 500 °C for 1 h with a heating rate of 10 °C/min. Unlike the first product prepared by using nickel chloride, the final powder showed larger particle sizes ranging between 20 and 100 nm, as shown in Figure 22a–c.
(iii) The solution was prepared by dissolving 5.2 mmol of nickel sulfate in 80 mL of distilled water, to which 0.083 mol of urea was added. The mixture was then heated to 80 °C for a duration of 4 h. The green precipitate was filtered, thoroughly washed with distilled water and ethanol, and dried at room temperature to obtain nanopowder with an agglomerated hexagonal sheet-like morphology.

As the surface morphology significantly affects the sensing performance, one can see the high response of the first powder whose nanoparticles are stacked in a porous structure. The very fine dimensions increase the surface-to-volume ratio which can enhance the sensitivity to formaldehyde with low concentrations (107–532 ppb) at 200 °C.

NiO nanoparticles with a size of 23 nm, prepared by Sol-Gel and deposited on the ITO substrate by dip-coating, showed a high selectivity to low concentrations of NO_2_ at 150 °C. The solution was prepared by dissolving nickel acetate in ethylene glycol monomethyl ether and stirring for 3 h at room temperature. Ammonia was added dropwise to the solution under stirring for 2 h at 60 °C. Then, it was aged at room temperature for 24 h before dip-coating at a rate of 500 µm/s and for 30 s.

- **Co_3_O_4_**

**Figure 23:**
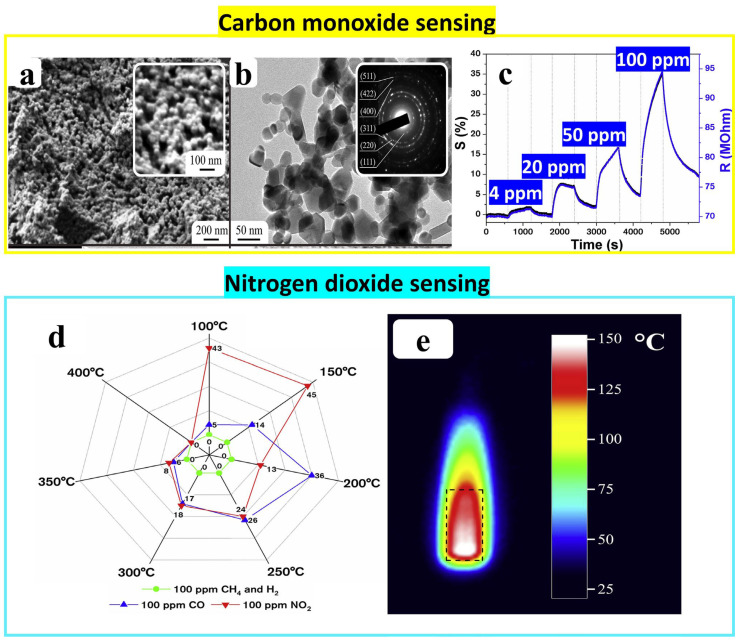
Sensing properties of cobalt oxide to CO: (**a**) SEM image (inset: zoom), (**b**) TEM image (inset: SAED pattern) and (**c**) sensitivity to CO at different gas concentrations at 200°C. Cobalt oxide as NO_2_ sensor: (**d**) sensing response to 100 ppm of H_2_, CH_4_, CO and NO_2_ at different working temperatures, (**e**) thermogram of the receptor layer surface when detecting the maximum response to NO_2_. Reprinted/adapted with permission from Ref. [70]. 2020, Elsevier.

To prepare cobalt oxide nanostructures, a solution of cobalt acetylacetonate and butyl alcohol was heated in a flask under reflux conditions and stirred while being subjected to heat treatment in a glycerin bath at 145 °C. During this process, ethanol and deionized water were poured into the solution while stirring to form the gel which was dried at 120 °C and then calcined at high temperature. The partial substitution of chelate ligands by alkoxy groups resulted in the formation of a hydrolytically active hetero–ligand complex [Co(C_5_H_7_O_2_)_2−x_(C_4_H_9_O)_x_] that was used as functional ink for plotting and printing on the Pt/Al_2_O_3_/Pt substrate at a speed of 50 mm/min and resolution of 10 lines/mm. It is worth noting that the gel was heated at 500 °C for 1 h, resulting in a Co_3_O_4_ cubic spinel-type structure with single-phase crystallinity. The film was composed of porous agglomerates, with an average pore size of 38 ± 4 nm. These agglomerates were constructed from spherical particles, each with an average size of 37 ± 4 nm. The gas testing of the cobalt oxide film showed a selectivity to 100 ppm of CO and NO_2_ with high responses at operating temperatures of 200 °C and 150 °C, respectively [70] (see Figure 23d).

- **CuO**

**Figure 24:**
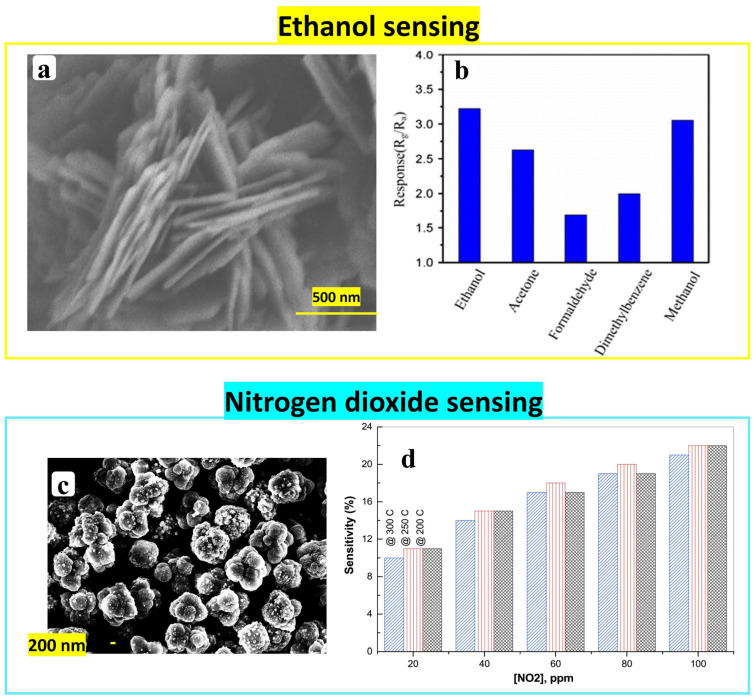
Sensitivity of CuO nanostructure to ethanol: (**a**) SEM image of CuO nanosheets and (**b**) selectivity measurement to different gases. Reprinted/adapted with permission from Ref. [73]. 2016, Springer Nature. CuO as NO_2_ sensor: (**c**) SEM image of CuO particales and (**d**) sensitivity measurements to different concentrations of NO_2_ at different operating temperatures (200 °C, 250 °C, and 300 °C). Reprinted/adapted with permission from Ref. [72]. 2021, Elsevier.

Wang et al. [71] demonstrated CuO sensing properties of 1 ppm for reducing volatile organic compounds at 220 °C. The derived Sol-Gel material showed the morphology of nanoparticles with a size of approximately 100 nm, obtained by dissolving 1 g of copper acetate in 15 mL of iso-propyl alcohol, mixed with 1 mL of the stabilizer of ethanolamine under continuous stirring at 80 °C for 2 h. The mixture was cooled down to room temperature and maintained for 24 h. The obtained material was calcined at 500 °C for 2 h, giving a wrinkle-like surface devoted to detecting low levels of reducing gases. This morphology can enlarge the contact area with air. Additionally, the wrinkles were composed of particles and micropores that can provide more contact area for the gas with the interior material; hence, the adsorption–desorption reactions will increase. Despite the fast response/recovery times (12 s/8 s: acetone, 13 s/13 s: methanol, and 13 s/9 s: ethanol), the CuO sensor is not selective (see Figure 24b) [71].

Cupric oxide also showed its sensitivity to oxidizing gas (NO_2_) without the selectivity test [72]. Figure 24c displays a CuO nanostructure (clusters of nanoparticles with spherical shape and size of 25–30 nm) with optimum operating temperature at 250 °C for NO_2_ sensor. The powder synthesis was based on adding 100 mL of ascorbic acid as the aqueous solution dropwise to the cupric acetate until reaching a molar ratio of 2:1 (ascorbic acid: Cu^2+^), under stirring for 30 min at room temperature. The change in the solution color from blue to reddish brown indicates the formation of metallic particles (Cu). The precipitate was filtered, washed with distilled water, and dried at 80 °C for 4 h. Then, the Cu_2_O/Cu compound was ground to fine powder and pelletized, followed by calcination at 600 °C for 24 h to obtain the final CuO material.

- **Moisture effect**

Metal oxide semiconductor sensors are sensitive to water vapor, and the stability of these sensors is significantly affected by the ambient humidity levels. Water vapor is acknowledged as an inevitable and critical interfering gas [74,75,76]. The sensor surface has available adsorption sites that can be filled by H_2_O molecules [77]. Degler et al. [77] examined three distinct approaches to water adsorption, focusing on the interplay between water vapor and SnO_2_. The surface composition, sensor resistance, and various pathways of water adsorption can influence the gas-sensing properties [78,79]. Surface modification results from the adsorption of water molecules influencing the adsorption behavior of various gases [80,81,82]. Cristian. E. Simion et al. [83] explored the reactivity of nickel oxide to CO and H_2_ under varied environmental conditions, employing diffuse reflectance infrared Fourier transform spectroscopy (DRIFTS). Their findings indicated a marginal elevation in sensor signals for hydrogen when exposed to ambient humidity. This is attributed to the non-interference between H_2_ and H_2_O for the same sites, as observed on SnO_2_ [84]. The baseline resistance of the studied NiO material remains largely unaffected by water vapor. Unlike many commonly used materials, the primary reaction of water vapor involves filling oxygen vacancies with OH groups. This characteristic sets the material apart. In contrast, on SnO_2_, the interaction between water and reducing gases for specific adsorption sites, dependent on temperature, results in a notable decline in sensor signals under humid conditions [77,85].

### 4.6. Tunability of MOXs

Tuning the MOX properties by doping or combining different materials, to achieve synergistic effects, can enhance sensing performance in various ways. Composites of metal oxides and doped metal oxides are two distinct approaches differing in terms of their composition and how they function as gas sensors. It is worth mentioning that Sol-Gel is considered a simple, cost-effective, and widely used method to combine and dope materials.

- **Doping**

**Figure 25:**
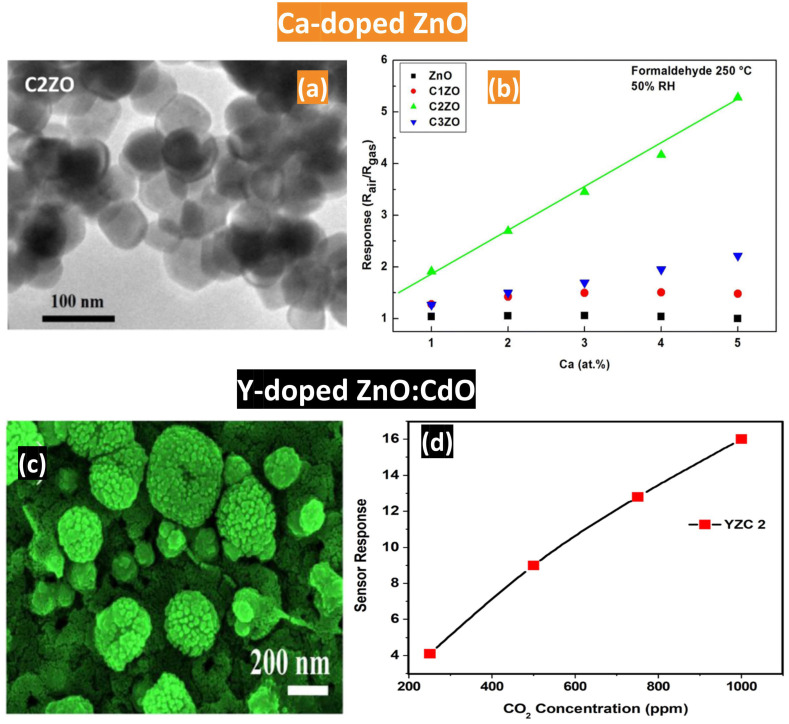
Doping effects on gas sensitivity of Sol-Gel-derived metal oxides: Ca-doped ZnO: (**a**) TEM image of nanoparticles and (**b**) gas response of pristine ZnO and Ca-doped ZnO to formaldehyde at 250 °C. Reprinted/adapted with permission from Ref. [86]. 2020, Springer Nature. Y-doped ZnO:CdO: (**c**) SEM image of cauliflower-like morphology and (**d**) gas response to different concentrations of carbon dioxide. Reprinted/adapted with permission from Ref. [87]. 2021, Elsevier.

Dopants induce changes in the microstructure, morphology, activation energy, and band gap of MOXs, thereby augmenting their capabilities for gas detection. The incorporation of dopants can lead to the formation of oxygen vacancies in these semiconductors, which, in turn, increases the adsorption sites and enhances their gas responsiveness. Figure 25 displays the doping effects on the morphology and gas response in ZnO and ZnO:CdO materials.

Hou et al. [88] prepared ZnO and Ga-doped ZnO materials using zinc acetate and gallium nitrate precursors, with a granular and dense surface where the grain size decreased with the doping concentration varying from 0.1 at.% to 1 at.%. The sensor response to hydrogen reached its highest value at 130 °C for the doping value of 0.3 at.%. This result can be attributed to a collective interplay of various factors, including grain size, crystallinity, defect concentration, and the presence of impurities. However, an excessive doping level showed a detrimental effect on ZnO performance despite its smallest grain size. The authors explained this degradation by the possible formation of a second phase of the alloyed compound where the Ga dopants could not form a solution with the host metal oxide. Another dopant (Ca) was used in tuning the ZnO properties for formaldehyde detection, as shown in Figure 25a,b [86]. Calcium-doped ZnO nanoparticles exhibited a higher response compared to the pure material with an optimum calcium concentration of 2 at.%.

Choudhary et al. [87] studied the yttrium doping effect on ZnO:CdO nanocomposite properties for carbon dioxide sensing. They used zinc acetate di-hydrate and cadmium acetate di-hydrate as the precursor, 2-methoxyethanol as the solvent, and mono ethanolamine as the stabilizer. The yttrium-to-ZnO:CdO loading ratio was maintained at 0, 0.5, 1, and 1.5 at.%, resulting in different morphologies (see Figure 25c,d). The cauliflower-like structure with 0.5 at.% showed the most significant response to CO_2_ owing to the extensive specific surface area that provides for more interactions with CO_2_ molecules. Additionally, the Y-dopant (0.5 at.%) demonstrated the lowest working temperature (27 °C) when compared to other Y-dopants (1 and 1.5 at.%) with operating temperatures of 50 °C and 100 °C, respectively.

- **Composites**

Composites of metal oxides involve combining MOX with other materials, such as polymers, carbon-based materials, binary selenides, binary sulfides, or other metal oxides, to create a hybrid material with improved gas sensing properties. This combination allows for enhanced gas responsiveness, offering advantages like improved mechanical stability, tunable characteristics, enhanced selectivity, and reduced operational temperature. By strategically selecting the secondary material in the composite, specific functionalities can be optimized, such as increasing the surface area for gas adsorption or enhancing electrical conductivity, making composites of metal oxides a versatile and effective solution for gas sensing applications.

Composites can form n-n, p-p, or n-p heterojunctions that play a significant role in optimizing the performances of MOX sensors.

**Figure 26:**
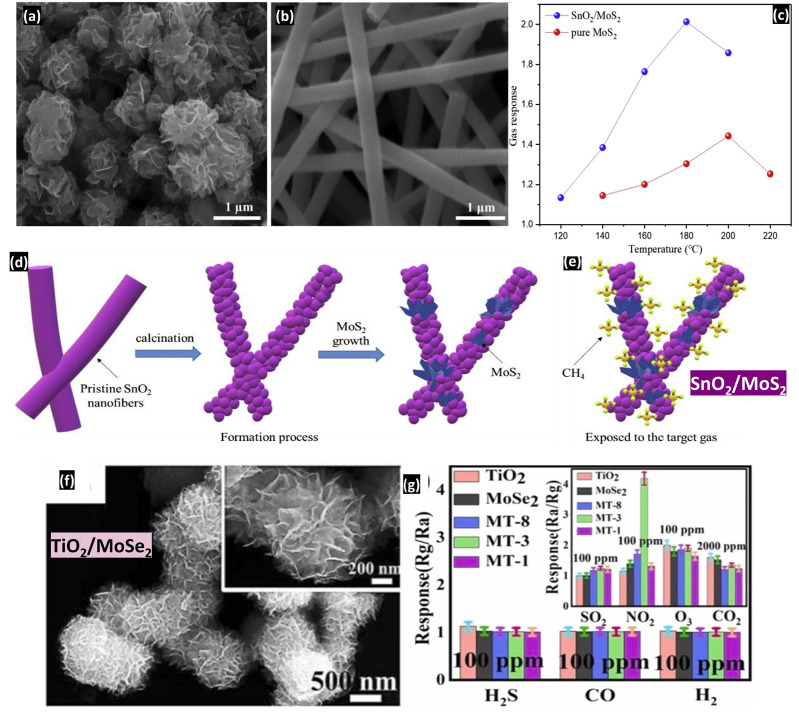
MOX-based composites prepared by Sol-Gel process for gas sensing applications: SnO_2_-MoS_2_: SEM images of (**a**) pure MoS_2_ and (**b**) pure SnO_2_, (**c**) gas response to CH_4_ of MoS_2_ and SnO_2_-MoS_2_ at different operating temperatures, schematic illustration of growth of pristine SnO_2_ nanofibers and their decoration with MoS_2_ (**d**) before and (**e**) after exposure to gas. Reprinted/adapted with permission from Ref. [90]. 2019, Elsevier. TiO_2_-MoSe_2_: (**f**) SEM images of TiO_2_-MoSe_2_ nanoflowers (inset: zoom) and (**g**) selectivity measurements of pristine TiO_2_ and MoSe_2_, and their composites to different gases. Reprinted/adapted with permission from Ref. [91]. 2023, Elsevier.

An example of an n-n composite heterojunction (ZnO/SnO_2_) is reported in the following paragraph [89] and another example of an n-n material SnO_2_-MoS_2_ [90] is presented in Figure 26a–e.

The preparation of a ZnO/SnO_2_ thick film by Sol-Gel was based on dissolving tin oxide in nitric acid and adding it to zinc nitrate under continuous stirring at 80 °C with a molar ratio of Zn:Sn = 2:1. Following several minutes of stirring, citric acid with a molar ratio of 1:1.5 and polyethylene glycol were introduced into the solution under stirring until forming the sol that was calcined at 400–800 °C in air for 4 h and then annealed at 600 °C for 4 h, resulting in the ZnO/SnO_2_ compound. The enhanced acetone sensing performance of nanocomposites compared to their individual constituents was attributed to the n-n heterostructure established at the interface of ZnO and SnO_2_. Also, the optimum temperature to detect 0.5 ppm of acetone was 180 °C for the composite compound, 200 °C for ZnO, and 220 °C for SnO_2_, which was dependent on the varying equilibrium between the adsorption and desorption of the gas molecules on the sensing film’s surface [89].

The second example of the n-n heterostructure is composed of tin dioxide and molybdenum disulfide: SnO_2_-MoS_2_ [90].

SnO_2_ nanofibers were prepared by dissolving 1.128 g of tin chloride in a mixture of absolute ethanol (5 mL) and N,N-Dimethylformamide (5 mL). After adding PVP, the solution remained under stirring for 6 h at 50 °C and was then used as a precursor for the electrospinning of tin dioxide nanofibers at a rate of 1 mL/h. After being annealed at 500 °C for 3 h, 0.004 g of SnO_2_ nanofibers was added to 0.038 g of thioacetamide and 0.048 g of sodium molybdate to be dispersed in 30 mL of deionized water under vigorous stirring for 1 h. The hydrothermal process assisted the Sol-Gel preparation by putting the solution in a 50 mL autoclave at 220 °C for 24 h. The flower-like molybdenum disulfide was synthesized following the last process without adding SnO_2_ nanofibers. The combination of these two materials resulted in the decoration of the surface of SnO_2_ porous nanofibers by nanoflowers of MoS_2_ with an improved sensitivity to CH_4_ gas and a reduced operating temperature when compared to the pure MoS_2_ sensor. The interface within the MoS_2_/SnO_2_ nanocomposite heterostructure plays a pivotal role in enhancing the sensor’s gas response. This enhancement stems from the electron transfer occurring when SnO_2_ and MoS_2_ come into contact. The MoS_2_ nanoflowers transfer electrons to the SnO_2_ nanofibers due to the higher work function of MoS_2_ (Φ = 4.70 eV) compared to SnO_2_ (Φ = 4.52 eV) and the stronger electron affinity of SnO_2_. This heterojunction effect continues until their Fermi energies are in equilibrium. Moreover, this process promotes the adsorption of oxygen species, facilitating the release of electrons back into the conduction band when exposed to CH_4_. Consequently, the carrier concentration within the heterojunction increases, leading to heightened conductivity in the MoS_2_/SnO_2_ nanocomposite [90].

Regarding the p-n heterostructures, we start by describing the rGO-loaded ZnO compound. Zain Ul Abideen et al. [92] reduced graphene oxide using 10 mL of hydrazine monohydrate and then mixed it with deionized water to prepare an rGO suspension to which they added the mixture of zinc acetate and the aqueous solution of PVA, obtained after stirring for 10 h. To make a viscous solution, the mixture was maintained under vigorous stirring for 1 h and then used in electrospinning rGO-loaded ZnO on the SiO_2_/Si substrate at a rate of 0.03 mL/h, followed by annealing in air at 600 °C for 30 min. The electrospun composite showed a response to 5 ppm of CO at 400 °C higher than that of ZnO nanofibers.

The polycrystalline ZnO nanofibers demonstrate their sensitivity to CO where the potential barrier height is reduced at the interface of the nanograins (ZnO/ZnO) under exposure to the gas species. Consequently, the resistance decreases along the grain boundaries, leading to measuring the gas response. This response is proved to be bolstered when loading rGO nanosheets on the ZnO nanofibers due to the high specific surface area that rGO can provide, increasing the adsorption sites for gas species. In addition, combining rGO with ZnO induces numerous n-p heterojunctions where electrons migrate from ZnO to rGO, establishing a balanced p-n heterointerface. This signifies that the rGO nanosheets function as electron acceptors, leading to an expansion of the depletion layer within the ZnO nanofibers. When CO gas molecules interact with oxygen species previously adsorbed on the ZnO surfaces, it is anticipated that the expanded depletion layer will exhibit even greater variations; therefore, an increase in sensing response is observed.

TiO_2_-MoSe_2_ also represents p-n type heterostructures prepared by Yang et al. [91] for NO_2_ gas detection.

On the one hand, TiO_2_ quantum dots were made using the Sol-Gel method. A mixture comprising cyclohexane, ethanol, and 12 mL of butyl titanate was stirred for 10 min, heated to reflux, and maintained for 10 h, resulting in the formation of a light-yellow solution. An amount of ethanol was gradually added to the solution until achieving a white suspension.

On the other hand, MoSe_2_ nanosheets were obtained using the solvothermal-assisted Sol-Gel process. A mixture of 0.4 g of selenium powder and a 30 mL solution comprising ethanol and water was stirred. Subsequently, 0.6 g of sodium molybdate was added to the solution and subjected to ultrasonic treatment. An amount of 94 mg of sodium borohydride was incorporated, resulting in the solution transitioning from a dark gray to a reddish-brown color. Following this, the solution was placed in a 50 mL autoclave and maintained at 200 °C for 48 h. Finally, the products were obtained by thoroughly rinsing with ethanol and water three times, and subsequently, the material was dried at 60 °C.

Various MoSe_2_ contents were introduced into a mixture of ethanol and water under stirring. Following this, ultrasonic treatment was applied for 2 h. In parallel, 40 mg of TiO_2_ powder was dissolved in water and subsequently blended with MoSe_2_ through stirring for an additional 2 h to yield the TiO_2_-MoSe_2_ composites with different molar ratios.

It is worth mentioning that TiO_2_ has the lowest gas response due to its poor conductivity [91]. However, the combination between TiO_2_ and MoSe_2_ enhances the responsiveness to 100 ppm of NO_2_ gas at 25 °C where the highest value is obtained at Ti:Mo = 1:3 (see Figure 26f,g). The authors attributed this enhancement to the formation of p-n heterojunctions (type II) within the composites. The difference in work function results in the migration of electrons from MoSe_2_ to the conduction band of TiO_2_, while holes from TiO_2_ migrate to the valence band of MoSe_2_. Consequently, more free electrons accumulate in the TiO_2_ conduction band, providing additional active sites for the adsorption of gas species, hence changing the resistance of the composites.

Another suggestion to boost the TiO_2_ sensing response is mentioned in reference [93]. Wang et al. [93] dispersed Sol-Gel-derived TiO_2_ on amorphous carbon and tested its sensing response to 10 ppm of NO_2_ at room temperature: 1.8 for pure TiO_2_ vs. 2.4 for the TiO_2_–carbon composite. The improved sensitivity is due to the high adsorption capacity of the TiO_2_–carbon compound (the oxygen adsorption is increased) and the formation of p-n heterojunctions. Common knowledge dictates that, at room temperature, when oxygen adheres to a semiconductor’s surface, O_2_^−^ species will be created, leading to an electron-depleted layer that will be thicker within the TiO_2_–carbon composite, hence increasing its resistance.

The continuous advancement in the Sol-Gel method has led to innovative techniques and modifications, further enhancing its efficiency and applicability. Some notable recent advancements include templated Sol-Gel synthesis where the use of templates, such as micelles or nanotubes, allows precise control over the nanostructure’s shape and dimensions, and facilitates the fabrication of complex metal oxide architectures [12,94,95]. Other processes such as hydrothermal [96], solvothermal [97], auto-combustion [98], microwave, or ultrasonic treatments [99] can also assist the Sol-Gel synthesis of metal oxides, resulting in obtaining hierarchical structures that combine different metal oxides with high specific areas and doping them to optimize their gas sensitivities. We summarize pure, doped, and composite metal oxides with different responses/sensitivities to various gases in Table 1, which is confined to the recent research published since 2022, reporting the sensing performance of Sol-Gel-derived MOX sensors to inflammable and highly toxic gases.

### 4.7. Outlook and Perspectives

Climate change is reshaping our world, impacting different regions. In the future, the adverse effects are likely to intensify significantly. The primary challenge is to minimize VOCs and other gas emissions. A crucial step in addressing this issue involves precisely measuring and detecting low levels of such compounds in the Earth’s atmosphere. To achieve this, we need efficient measurement systems equipped with cost-effective, robust, accurate, and innovative sensing devices. The field of sensors is rapidly evolving, with MOX materials gaining the most attention due to their ability to fulfil all these requirements. Sol-Gel stands out as a cost-effective and environmentally friendly approach for producing metal oxide nanostructures, as it often involves water-based solutions and avoids the use of hazardous chemicals. Its advantages include low-temperature requirements, precise control over size and morphology, and the ability to create materials with high surface areas to enhance the chemical interactions between gas species and the sensing surface. The ease of introducing other experimental approaches like hydrothermal, solvothermal, microwave treatment, and annealing is another option provided by the Sol-Gel technique. These experiments allow the synthesis of hierarchical structures with a high surface-to-volume ratio, which boosts the gas–surface interactions and sensitivity. Among issues that MOX sensors face since their discovery, we report selectivity. By changing the operating temperature, researchers can tune the selectivity of sensors due to the variation in the adsorption/desorption effect and charge transfer with temperature. Additionally, Sol-Gel offers the accessibility to enhance selectivity by doping metal oxides and combining them in composites to further enhance their responsiveness against gases and to produce more selective sensors at lower operating temperatures, which is challenging for MOX production to reach low-power consumable devices. Sol-Gel-derived metal oxides hold great promise for a wide range of sensing applications in the future. To enhance their sensing properties, researchers should focus on nanostructuring, doping, surface functionalization, and composite materials. The tailored development of materials for specific applications, a commitment to green synthesis, IoT integration, and the use of advanced technologies such as machine learning will contribute to the continued advancement of metal oxide sensors in the years to come.

However, it is essential to acknowledge certain limitations for such synthesis methods, such as the sensitivity to reaction conditions, necessitating careful optimization, and the requirement for post-processing steps to achieve the desired material properties. Despite these challenges, the Sol-Gel method remains a versatile and promising avenue for the tailored fabrication of MOX nanostructures with diverse applications in catalysis and sensing.

## 5. Conclusions

Metal oxide gas sensors offer several benefits compared to alternative solid-state gas monitoring systems, including affordability, ease of production, and compact design. Nevertheless, achieving high-sensitivity characteristics in these sensors is a notable challenge, primarily due to the critical influence of the shape and structure of sensing materials. This presents a significant hurdle in optimizing the gas sensing properties of materials or dense films. Sol-gel technology, a six-decade-old method, offers an alternative to traditional thin-film deposition techniques like chemical vapor deposition, molecular beam epitaxy, and sputtering. These thin films are becoming increasingly popular in various applications, including gas sensors, due to their low-temperature fabrication, controlled structural morphology, high purity, and enhanced sensitivity.

Sol-Gel processing yields materials with a high specific surface area and increased porosity by adjusting the precursors’ nature and concentration, solvents, molar ratio, solution pH, aging period, and bath temperature. Another important step is the post-heat treatment that promotes the poly-condensation and crystallization of the gel and improves its mechanical properties and structural stability. Some experimental evidence suggests lower calcination temperatures to ensure higher porosity and avoid agglomeration and crystal phase transition. Low-dimensional materials (nanoparticles, nanowires, nanosheets, hierarchical microstructures) are achievable using Sol-Gel and highly recommended for greater specific surface areas, which elevates the adsorption sites to provide better gas sensing. Gas response, sensitivity, and stability are the main factors impacting the MOX sensor efficiency where the response of n-type and p-type materials to various gases at different temperatures was shown in this review. These parameters can be enhanced through Sol-Gel by combining different metal oxides in composites, leading to a higher surface reactivity and carrier transfer between heterogeneous junctions. The synergistic effect is also credited for the improvement in gas sensing of MOX composites by enhancing the catalytic activity and increasing the resistance modulation. Sol-Gel also enables the doping strategy to modify the MOX parameters and enhance the gas responsiveness as reported in this review. The distinction between the effects of doping and forming heterojunctions is emphasized, noting that a heterojunction can be advantageous for carrier separation and larger depletion layers, while doping provides more adsorption sites and charge carriers during gas–surface interactions.

## Figures and Tables

**Table 1:** Summary of the response of Sol-Gel-derived VOC sensors reported since 2022.

Target Gas	Sol-Gel Material	Gas Concentration-Operating Temperature (°C)	Response/Sensitivity
Nitrogen dioxide	NiO [64]	25 ppm-175 °C	1.68 *
	CeO_2_ [64]	25 ppm-200 °C	1.93 *
	CaCu_3_Ti_4_O_12_ [100]	100 ppm-250 °C	~7 *
	CeO_2_-NiO [64]	25 ppm-125 °C	3.06 *
	TiO_2_ [91]	100 ppm-RT	1.324 **
	MoSe_2_ [91]	100 ppm-RT	1.705 **
	TiO_2_-MoSe_2_ (Ti:Mo 1:8) [91]	100 ppm-RT	4.16 **
	TiO_2_-MoSe_2_ (Ti:Mo 1:3) [91]	100 ppm-RT	1.31 **
	TiO_2_-MoSe_2_ (Ti:Mo 1:1) [91]	100 ppm-RT	1.15 **
	InZnO (1:8 as In:Zn ratio) [101]	1 ppm-RT	18% ^NA^
	InZnO (16:1 as In:Zn ratio) [101]	1 ppm-RT	−30% ^NA^
	CuFe_2_O_4_ [102]	5 ppm-260 °C	1.45 *
	TiO_2_ [103]	1 ppm-RT	22 **
	N719-sensitized TiO_2_ [103]	1 ppm-RT	109 **
	N719-sensitized POM/TiO_2_ [103]	1 ppm-RT	231 **
	Fe_2_O_3_ [104]	10 ppm-350 °C	4.2 *
	0.25CuO-0.75Fe_2_O_3_ [104]	10 ppm-350 °C	7.1 *
	0.5CuO-0.5Fe_2_O_3_ [104]	10 ppm-350 °C	8.8 *
	0.75CuO-0.25Fe_2_O_3_ [104]	10 ppm-350 °C	5.8 *
	CuO [104]	10 ppm-350 °C	1.8 *
Sulfur dioxide	CuFe_2_O_4_ [102]	5 ppm-260 °C	2 *
Ammonia	InZnO (16:1 as In:Zn ratio) [101]	1 ppm-RT	11% ^NA^
	Fe_3_O_4_ [105]	200 ppm-RT	30–40% ***
	Zn_0_._5_Fe_2_._5_O_4_ [105]	200 ppm-RT	60–70% ***
	ZnO/Co_3_O_4_/CuO [106]	20 ppm-RT	6.2 **
	CuO [107]	50 ppm-RT	27.5 *
Hydrogen sulfide	InZnO (16:1 as In:Zn ratio) [101]	1 ppm-RT	−7.4% ^NA^
	BaSnO_3_ [108]	1 ppm-150 °C	17 ****
	La-doped BaSnO_3_ [108]	1 ppm-150 °C	16.6 ****
	ZnO NCs/F-GaN [109]	50 ppm-220 °C	89.7 **
	Fe_0_._6_Al_1_._4_O_3_ [110]	100 ppm-200 °C	46.69% *****
	CuFe_2_O_4_ [102]	5 ppm-260 °C	1.33 *
	ZnO/La_0_._8_Sr_0_._2_FeO_3_ [111]	1 ppm-200 °C	32.97% *****
	Fe_2_O_3_ [104]	1 ppm-350 °C	3 **
	0.25CuO-0.75Fe_2_O_3_ [104]	1 ppm-350 °C	10.6 **
	0. 5CuO-0.5Fe_2_O_3_ [104]	1 ppm-350 °C	15.3 **
	0.75CuO-0.25Fe_2_O_3_ [104]	1 ppm-350 °C	4.3 **
	CuO [104]	1 ppm-350 °C	2.4 **
Hydrogen	Pt-doped WO_3_ [112]	10,000 ppm-RT	6.175% ^×^
	MnCo_2_O_4_ [113]	250 ppm-160 °C	<4% ^NA^
	Al0._075_Cd_0_._15_Zn_0_._225_Ni_0_._55_Fe_2_O_4_ [114]	25 ppm-200 °C	66.95 ^×^
Acetone	CuO [107]	50 ppm-RT	1.2 *
	CuFe_2_O_4_ [102]	5 ppm-260 °C	0.44 *
	ZnO [115]	100 ppm-RT	11.78% ^NA^
	GO/ZnO [115]	100 ppm-RT	31.17% ^NA^
	Co_3_O_4_ [116]	20 ppm-225 °C	0.04 *
	0.2 GO-Co_3_O_4_ [116]	20 ppm-225 °C	9.26 *
	Cu/G-doped ZnO [117]	50 ppm-270 °C	3.21 **
	ZnO [118]	10 ppm-450 °C	25 *
	SnO_2_ [118]	10 ppm-350 °C	<20 *
	TiO_2_ [118]	10 ppm-350 °C	<30 *
	Fe-doped rGO decorated WO_3_ [119]	10 ppm-130 °C	4.678 **
	LaFeO_3_ [120]	100 ppm-100 °C	132.1 *
	LaFeO_3_/La_2_O_3_ [120]	100 ppm-100 °C	25.7 *
	LaFeO_3_/Fe_2_O_3_ [120]	100 ppm-100 °C	47.1 *
	Bi_0_._99_Ca_0_._01_FeO_2_._995_ [121]	100 ppm-220 °C	4.6 *
Carbon monoxide	InZnO (16:1 as In:Zn ratio) [101]	1 ppm-RT	−1.7% ^NA^
	ZnO [118]	50 ppm-450 °C	<2 *
	SnO_2_ [118]	50 ppm-450 °C	<7 *
	TiO_2_ [118]	50 ppm-450 °C	<4 *
	CuFe_2_O_4_ [102]	5 ppm-260 °C	1.22 *
	SnO_2_ [122]	470 ppm-450 °C	149.9 ****
Carbon dioxide	Gd-ZnO/TiO_2_[123]	50 ppm-50 °C	93.4% ***
	SnO_2_ [124]	NA-30 °C	78.57% *****
	Sb(1.8at%)-doped SnO_2_ [124]	NA-80 °C	18.74% *****
	Sb(2.4at%)-doped SnO_2_ [124]	NA-80 °C	71% *****
	Ni(10 mol.%)ZnO [125]	40 ppm-RT	33.3% *****
	Ni(20 mol.%)ZnO [125]	40 ppm-RT	61.1% *****
	Ni(30 mol.%)ZnO [125]	40 ppm-RT	16.6% *****
	LaFeO_3_ [126]	5000 ppm-220 °C	1.72% *****
	LaCo_0_._1_Fe_0_._9_O_3_ [126]	5000 ppm-220 °C	6.69% *****
Triethylamine	SnO_2_ [127]	100 ppm-325 °C	7.5 **
	SnO_2_/SiO_2_ [127]	100 ppm-280 °C	18 **
	CuO [107]	50 ppm-RT	140 *
	Co_3_O_4_ [128]	100 ppm-130 °C	3.07 *
	LaMnO_3_/Co_3_O_4_ [128]	100 ppm-130 °C	28.48 *
Trimethylamine	CuO [107]	50 ppm-RT	30.2 *
Liquified petroleum gas	CoFe_1.9_La_0.1_O_4_ [129]	0.9 ppm-RT	98% ***
Ethanol	CuFe_2_O_4_ [102]	5 ppm-260 °C	0.83 *
	Cu/G-doped ZnO [117]	50 ppm-270 °C	4.03 **
	HfO_2_ [130]	2000 ppm-RT	3.7 ^×^
	LaFeO_3_ [120]	100 ppm-100 °C	127.3 *
	LaFeO_3_/La_2_O_3_ [120]	100 ppm-100 °C	32.6 *
	LaFeO_3_/Fe_2_O_3_ [120]	100 ppm-100 °C	42.8 *
	CuO [107]	50 ppm-RT	6.9 *
	YFeO_3_ [131]	100 ppm-350 °C	18 *
Methanol	CuO [107]	50 ppm-RT	1.4 *
	Cu/G-doped ZnO [117]	50 ppm-270 °C	2.69 **
	M-LaFeO_3_ [132]	100 ppm-160 °C	8.9 *
	S-LaFeO_3_ [132]	100 ppm-160 °C	4 *
	ZnO/Co_3_O_4_/CuO [106]	100 ppm-RT	3.6 **
	LaFeO_3_ [120]	100 ppm-100 °C	67.5 *
	LaFeO_3_/La_2_O_3_ [120]	100 ppm-100 °C	31.7 *
	LaFeO_3_/Fe_2_O_3_ [120]	100 ppm-100 °C	30.1 *
N-butanol	CuO [107]	50 ppm-RT	1.9 *
Isopropanol	Cu/G-doped ZnO [117]	50 ppm-270 °C	4.06 **
	LaFeO_3_ [120]	100 ppm-100 °C	126.3 *
	LaFeO_3_/La_2_O_3_ [120]	100 ppm-100 °C	22.7 *
	LaFeO_3_/Fe_2_O_3_ [120]	100 ppm-100 °C	44.5 *
N-propanol	Fe_2_O_3_ [120]	100 ppm-200 °C	~1.8 *
	La_2_O_3_ [120]	100 ppm-280 °C	<1.1 *
	LaFeO_3_ [120]	100 ppm-100 °C	258.4 *
	LaFeO_3_/La_2_O_3_ [120]	100 ppm-100 °C	~102.2 *
	LaFeO_3_/Fe_2_O_3_ [120]	100 ppm-100 °C	94.9 *
Isopropene	TiO_2_ [118]	10 ppm-400 °C	<25 *
Ozone	CuAlO_2_ [133]	5 ppm-RT	12.32 ****
	CuGaO_2_ [134]	5 ppm-RT	5.2 ****
Formaldehyde	CuFe_2_O_4_ [102]	5 ppm-270 °C	2.45 *
	LaFeO_3_ [120]	100 ppm-100 °C	32.5 *
	LaFeO_3_/La_2_O_3_ [120]	100 ppm-100 °C	30.4 *
	LaFeO_3_/Fe_2_O_3_ [120]	100 ppm-100 °C	15.5 *
	In doped-LaFeO_3_ [135]	100 ppm-125 °C	122 *
	NiO [136]	100 ppm-RT	3013.5 *
	Mn-doped NiO [136]	100 ppm-RT	12,593 *
Dimethylformamide	CuO [107]	50 ppm-RT	1.1 *
Xylene	Dy-doped NiO	100 ppm-200 °C	7.4 *

RT: room temperature; NA: information is not available; * S = R_g_/R_a_; ** S = R_a_/R_g_; *** S = R_a_ − R_g_/R_a_; **** S = R_a_ − R_g_/R_g_; ***** S = R_g_ − R_a_/R_a_; ^×^ S = I_g_ − I_a_/I_a_.

## Data Availability

Data sharing not applicable.
